# Metabolic Signatures of Surface-Modified Poly(lactic-*co*-glycolic acid) Nanoparticles in Differentiated THP-1 Cells Derived with Liquid Chromatography-Mass Spectrometry-based Metabolomics

**DOI:** 10.1021/acsomega.2c01660

**Published:** 2022-08-12

**Authors:** Mohammad A. Al-natour, Salah Abdelrazig, Amir M. Ghaemmaghami, Cameron Alexander, Dong-Hyun Kim

**Affiliations:** † Molecular Therapeutics and Formulation Division, School of Pharmacy, University of Nottingham, Nottingham NG7 2RD, U.K.; ‡ Centre for Analytical Bioscience, Advanced Materials and Healthcare Technologies Division, School of Pharmacy, University of Nottingham, Nottingham NG7 2RD, U.K.; § Immunology & Immuno-bioengineering Group, School of Life Sciences, Faculty of Medicine and Health Sciences, Queen’s Medical Centre, University of Nottingham, Nottingham NG7 2RD, U.K.; ∥ Division of Pharmaceutics and Pharmaceutical Sciences, Faculty of Pharmacy, University of Petra, Amman 11196, Jordan; ⊥ Department of Pharmaceutical Chemistry, Faculty of Pharmacy, University of Khartoum, Khartoum 11115, Sudan

## Abstract

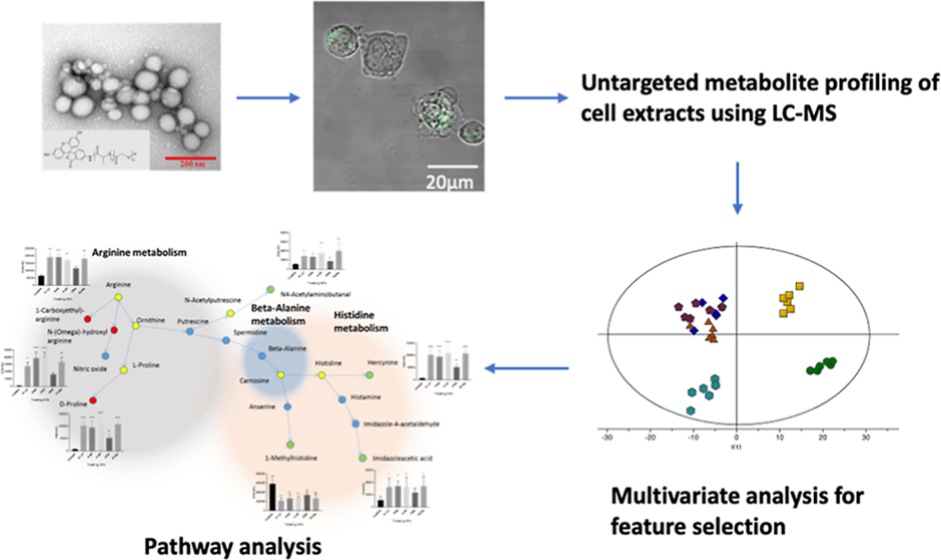

Polymeric nanoparticles (NPs) are widely used in preclinical drug delivery investigations, and some formulations are now in the clinic. However, the detailed effects of many NPs at the subcellular level have not been fully investigated. In this study, we used differentiated THP-1 macrophage cells, as a model, to investigate the metabolic changes associated with the use of poly (lactic-*co*-glycolic acid) (PLGA) NPs with different surface coating or conjugation chemistries. Liquid chromatography-mass spectrometry-based metabolic profiling was performed on the extracts (*n* = 6) of the differentiated THP-1 cells treated with plain, Pluronic (F-127, F-68, and P-85)-coated and PEG–PLGA NPs and control (no treatment). Principal component analysis and orthogonal partial least squares-discriminant analysis (OPLS-DA) in conjunction with univariate and pathway analyses were performed to identify significantly changed metabolites and pathways related to exposure of the cells to NPs. OPLS-DA of each class in the study compared to the control showed clear separation and clustering with cross-validation values of *R*^2^ and *Q*^2^ > 0.5. A total of 105 metabolites and lipids were found to be significantly altered in the differentiated THP-1 cell profiles due to the NP exposure, whereas more than 20 metabolic pathways were found to be affected. These pathways included glycerophospholipid, sphingolipid, linoleic acid, arginine and proline, and alpha-linolenic acid metabolisms. PLGA NPs were found to perturb some amino acid metabolic pathways and altered membrane lipids to a different degree. The metabolic effect of the PLGA NPs on the cells were comparable to those caused by silver oxide NPs and other inorganic nanomaterials. However, PEG–PLGA NPs demonstrated a reduced impact on the cellular metabolism compared to Pluronic copolymer-coated PLGA and plain PLGA NPs.

## Introduction

Polymers formulated as nanoparticles (NPs) are now in clinical use for delivery of anticancer drugs,^1^ and many further examples are under investigation for oncology applications and a variety of other disease indications.^2^ Poly(lactic-*co*-glycolic acid) or PLGA is one of the most widely used polymers in drug delivery as it is biodegradable, is well-tolerated in humans, and is readily formulated into microparticles, NPs, and monoliths for implantation.^3^ However, PLGA NPs are not suitable for systemic injection without a surface treatment to ensure colloidal stability, as NPs prepared from the native copolymer are prone to aggregation in aqueous media. Accordingly, PLGA formulations are often coated or derivatized with a hydrophilic or amphiphilic surface layer,^4^ or the lactide and glycolide comonomers are ring-opened with a hydrophilic polymer nucleophile, such as methoxy-poly(ethylene glycol), to generate an amphiphilic block copolymer.^5^ In general, these materials have been shown to be nontoxic in preclinical studies,^6^ and there is little evidence of any acute harmful effects to date.

However, hydrophilic polymers and polymeric amphiphiles are not necessarily inert, and there is some evidence that poly(ethylene glycol) (PEG) can be recognized by some antibodies,^7^ while copolymers of PEG with poly(propylene glycol) (PPG) have been shown to be membrane-active.^8^ These biological responses are not necessarily detrimental to therapeutic applications and indeed have been exploited to enhance delivery and efficacy in some ingenious ways,^9−11^ but nevertheless, further investigation of the activities of amphiphilic copolymers in cellular environments is required.

Various cell assays have been employed to assess the use of nanomaterials and their effect in relation to oxidative stress, inflammation, mitochondrial injury, and DNA and cell membrane damage; this is usually carried out by measuring the endpoint of cellular triggered toxicity by NPs.^12−14^ Nevertheless, the outcome from the single effect assays is not comprehensive and does not highlight the subtle biological response or the biochemical pathways affected by the exposure to NPs.^15^ Additionally, most of these conventional methods are colorimetric and prone to interference due to certain properties of specific NPs which can result in interference with the dyes or the enzymes in the assays.^16^ In particular, NPs containing labels or of certain sizes may absorb or scatter light during the colorimetric measurements, leading to inaccurate quantification.^17^ Hence, there is a pressing need to establish a new and robust in vitro method to assess the biological response of the NPs as drug carriers before their use in clinical applications.^16,18^

Transcriptomics and proteomics provide valuable information about cell phenotypes; however, such approaches typically do not capture the potential post-transcriptional or translational modifications which are critical in determining the cell function. On the other hand, metabolomics aims for the qualitative and quantitative measurement of the end products (i.e., metabolites) of the gene expression and the cellular metabolic activities in a biological system.^19,20^ The information gained using metabolomics allows the characterization of the metabolic phenotypes^21^ and therefore provides a valuable means to study the molecular effects and toxicity of polymeric NPs on the biological systems. Some studies have already been denoted for studying different NPs using metabolomics;^22−24^ however, in most cases, the studies were focused on the use of metal-based NPs for cosmetic and textile applications, and few have been focused on clinical use.

NPs can be recognized as foreign items in the body by the immune system. As a result, immune cells, particularly those involved in the innate immune response, which provide a quick, nonspecific reaction to potential threats, may recognize and neutralize them. As professional phagocytes, macrophages are an important cell component of the innate immunity. Macrophages are the initial line of defence against invading agents. In vivo studies have revealed that resident macrophages in the lungs, liver, and spleen are important in the clearance of NPs.^25^ NPs may interact with macrophages to affect not just their fate (e.g., NP clearance), but such an interaction could also trigger hazardous effects (e.g., inflammation and reactive oxygen species production).

Macrophages play a key role in the etiology of diseases including lung fibrosis and mesothelioma, which are caused by exposure to NPs and nanofibers.^26^ For instance, macrophages in the reticuloendothelial system and alveoli were found to engulf NPs after intravenous administration and inhalation, respectively.^27^ THP-1 cells that are differentiated into macrophages have been found to be a good model system for researching macrophage functions in vitro.^28^ In addition, previous studies have shown that mass spectrometry (MS)-based metabolite profiling was sensitive enough to detect subtle changes in intracellular metabolites between naïve and polarized macrophages.^29^ Therefore, in this work, liquid chromatography–MS (LC–MS)-based metabolite profiling was employed to assess the cellular metabolic response in human macrophages (differentiated THP-1 cells) after exposure to five different types of PLGA NPs with different surface functionalities.

## Experimental Section

### Chemicals and Materials

Lactide, glycolide (99%), benzyl alcohol, PEG methyl ether (*M*_n_ = 5000 g/mol), 5-aminofluorescein, tin(II) 2-ethylhexanoate (Sn(Oct)_2_, 92.5–100.0%), deuterated chloroform (CDCl_3_), RPMI 1640 medium, heat-inactivated fetal bovine serum (FBS), penicillin, streptomycin, l-glutamine, phorbol 12-myristate 13-acetate, and formaldehyde were purchased from Sigma-Aldrich (Gillingham, UK). Ammonium carbonate, isopropanol, and acetonitrile were of LC–MS grade, dichloromethane, methanol, diethyl ether, and acetone were of HPLC grade, and they were obtained from Fisher Scientific (Loughborough, UK). Authentic standards were prepared in five different mixtures (see full details in Table S1) at a concentration of 20 μM and coanalyzed with the samples for the identification of the metabolites in this study. The authentic standards were obtained from either Fisher Scientific (Loughborough, UK) or Sigma-Aldrich (Gillingham, UK) unless otherwise stated.

### Polymer Synthesis

PLGA, PEG–PLGA, and PLGA-5-aminofluorescein (PLGA-5AF) were synthesized by ring-opening polymerization of lactide and glycolide. The synthesis of these polymers was carried out using benzyl alcohol, and 5-aminofluorescein and PEG were used as initiators as follows

#### PLGA Synthesis

d,l-Lactide (4.32 g, 30 mmol) and glycolide (2.32 g, 20 mmol) were heated under nitrogen at 130 °C until the monomers had melted. Benzyl alcohol (0.108 g, 1 mmol) was added, and the reaction was carried out for 1 min. The catalyst SnOct_2_ (0.034 g, 0.5% w/w) was added, and the reaction continued for 3 h under nitrogen before being allowed to cool to room temperature The synthesized polymer was dissolved in dichloromethane and precipitated in diethyl ether three times to remove unreacted monomers and impurities. The precipitate of the polymer was filtered and dried under reduced pressure.

#### PEG–PLGA Synthesis

d,l-Lactide (3.03 g, 21 mmol), glycolide (1.63 g, 14 mmol), and MPEO (3.475 g, 0.695 mmol) were heated under nitrogen at 130 °C until the monomers and the initiator had melted. The catalyst SnOct_2_ (0.041 g, 0.5% w/w) was added, and the reaction continued for 3 h under nitrogen before being allowed to cool to room temperature. The synthesized copolymer was dissolved in dichloromethane and precipitated in ice-cold diethyl ether to remove unreacted monomers and other impurities, and the filtered precipitate was dried under reduced pressure.

#### PLGA-5AF

d,l-Lactide (4.32 g, 30 mmol), glycolide (2.32 g, 20 mmol), and 5-aminofluorescein (0.347 g, 1 mmol) were heated under nitrogen at 130 °C until the monomers and the initiator had melted. The catalyst SnOct_2_ (0.034 g, 0.5% w/w) was added, and the reaction continued for 3 h under nitrogen before being allowed to cool to room temperature. The synthesized copolymer was dissolved in acetone and precipitated in methanol six times to remove unreacted monomers, free dye, and other impurities. The filtered precipitate was dried under reduced pressure.

### Fabrication of PLGA NPs with Different Coating Chemistries

Plain, Pluronic-coated, and PEG–PLGA NPs were prepared by a standard nanoprecipitation technique with some modifications.^30^ A solution of PLGA (20 mg) and PLGA-5AF (5 mg) in acetone (10 mL) was added to 10 mL of the antisolvent (Milli-Q water) using a syringe pump (0.7 mL/min) to prepare PLGA NPs. By an analogous procedure, PEG–PLGA NPs were prepared using 12.5 mg of PEG–PLGA, 7.5 mg of PLGA, and 5 mg of the PLGA-5AF mixture. The stirring of the NP suspensions was continued at room temperature to ensure complete evaporation of acetone. The suspensions were filtered using 0.45 μm syringe filters to ensure sterility.

Three Pluronic poly(ethylene glycol)-*co*-poly(propylene glycol)-*co*-poly(ethylene glycol) (PEG–PPG–PEG) block copolymers were used for NP coating, namely, F-127 (12,200 g mol^–1^), F-68 (8400 g mol^–1^), and P-85 (4600 g mol^–1^). The prepared NPs were suspended in 0.1% w/v Pluronic water solutions for 24 h to ensure coating. Free Pluronics were removed by dialysis against 1 L of deionized water for 24 h using the 50 kDa membrane.

### Culture, Activation, and Treatment of THP-1 Cells with NPs

THP-1 cells (a human monocytic cell line) were cultured in T75 tissue culture flasks as previously described.^29^ The cells were cultured in RPMI media supplemented with 2 mmol/L l-glutamine, 100 μg/mL streptomycin, 100 U/mL penicillin, and 10% v/v heat-inactivated FBS at 37 °C under 5% CO_2_ and 95% relative humidity. To induce differentiation to macrophage-like cells, THP-1 cells were treated with phorbol-12-myristate-13-acetate (PMA) as previously described with minor modifications.^31^ THP-1 cells were then seeded in T25 flasks (6 million cells/flask) in RPMI media containing 50 ng/mL PMA for 24 h under the same starting culture conditions. After 24 h, the culture media was replaced with fresh starting media containing 100 μg/mL NPs. The cells were treated with the NPs for 24 h followed by metabolite extraction.

### Metabolite Extraction of the Differentiated THP-1 Cells Exposed to the Different NPs for LC–MS Metabolite Profiling

The differentiated THP-1 cells [control (no treatment), *n* = 6] and the differentiated THP-1 macrophage cells treated with F-127- (*n* = 6), F-68- (*n* = 6), P-85- (*n* = 6), and PEG (*n* = 6)-coated PLGA NPs and plain PLGA NPs (*n* = 6) were extracted for LC–MS analysis. The incubation media were removed, and the cells were, briefly, washed with phosphate-buffered saline (PBS) (37 °C); then, methanol (0.5 mL, −48 °C) was added to simultaneously quench the metabolism and extract the intracellular metabolites. The cells were scraped and vortexed vigorously for 1 h and centrifuged at 17,000 × *g* for 10 min (4 °C). The supernatants were removed and dried under vacuum at room temperature. The extracts were reconstituted in 70 μL of methanol (4 °C) and used for LC–MS. 10 μL from each sample was mixed and used as a pooled quality control (QC) sample to assess the instrument performance.

### Proton Nuclear Magnetic Resonance (^1^H NMR) Spectroscopy

^1^H NMR spectroscopy was performed at 400 MHz using a Brüker DPX 400 Ultrashield spectrometer (Coventry, UK). All the chemical shifts were acquired in ppm in reference to CDCl_3_. The spectra were analyzed using MestRENova 6.0.2 (Mestrelab Research, S.L., Santiago de Compostela, Spain).

### Particle Size and Zeta Potential Measurements

Mean hydrodynamic diameters and zeta potentials of the prepared NPs were determined using Malvern Zetasizer (Malvern Panalytical, Malvern, UK) equipped with 10 mW He–Ne laser operating at a wavelength of 633 nm by measuring the light scattering at 173° angle to incident radiation at 25 °C after diluting the samples with water.

### Transmission Electron Microscopy

The morphology of the prepared NPs was examined using transmission electron microscopy (TEM) (FEI Tecnai G2, Oregon, USA). NP suspensions in water (17 μL) were added onto a copper grid and wicked off after 10 min, and then, 3% w/v uranyl acetate was used as a negative stain prior imaging. The metabolic activity of the differentiated THP-1 cells after exposure to the NPs was observed.

The alamarBlue metabolic activity assay was used to assess the effects of the plain, Pluronic-coated, and PEG–PLGA NPs on the THP-1 cells (*n* = 3). The differentiated cells in 24-well plates were incubated with media containing 50, 100, 250, and 500 μg/mL of the different NPs for 24 h. The media was then removed, and the cells were washed with PBS and incubated (light-protected) in fresh media containing 10% alamarBlue for 3 h (37 °C). 100 μL of the spent media were transferred to 96-black-well plates, and the fluorescence was measured using an Optima FLUOstar plate reader at an excitation/emission of 540/580 nm.

### Cellular Uptake of NPs by Differentiated THP-1 Cells

The cellular uptake of the prepared NPs by the differentiated THP-1 cells was examined using confocal microscopy and flow cytometry as follows:

#### Confocal Microscopy

The THP-1 cells were differentiated on an eight-well chambered coverslip at 37 °C and 5% CO_2_ for 24 h. The media were replaced with fresh media (control) or media containing 100 μg/mL plain, Pluronic-coated, and PEGylated PLGA NPs for 1 h at 37 °C and 5% CO_2_. Following the treatment, the cells were washed twice with PBS and fixed with 4% formaldehyde in PBS (300 μL) for 15 min in the dark at room temperature. Subsequently, the plates were washed twice with PBS and stored with mounting media at 4 °C. The cells were imaged using a confocal laser scanning microscope (Zeiss LSM 700, Cambourne, UK) controlled with Zen software.

#### Flow Cytometry

The THP-1 cells were differentiated in 24-well plates at a seeding density of 500,000 cells/well for 24 h under the same conditions described for confocal microscopy. The media was replaced with fresh media (control) or media containing 100 μg/mL plain, Pluronic–coated, and PEGylated NPs for 3 h. After treatment, the cells were washed twice with PBS and detached using TrypLE Express Enzyme (Sigma-Aldrich, Gillingham, UK) for 30 min at 37 °C and 5% CO_2_ with occasional gentle rocking, after which TrypLE Express was deactivated by adding fresh media. The cells were then centrifuged and resuspended in fresh media before analysis in an FC 500 flow cytometer (Beckman Coulter, High Wycombe, UK). A total of 10,000 viable cells were gated for each analysis. Data were analyzed using Weasel software (Walter and Eliza Hall Institute for Medical Research, Melbourne, Australia).

### LC–MS for Untargeted Metabolomics

Chromatographic separation was performed on a ZIC-*p*HILIC column (5 μm, 4.6 × 150 mm, Merck Sequant, Watford, UK) maintained at 45 °C using an Accela UHPLC system (Thermo Fisher Scientific, Hemel Hempstead, UK) as previously described.^32,33^ Briefly, the mobile phases used were 20 mM ammonium carbonate in water (A), and 100% acetonitrile (B), and metabolites were separated by injecting 10 μL of the sample (4 °C) in a linear gradient of 300 μL/min of 20% A (0–15 min), 95% A (15–17 min), and 20% A (17–24 min).

MS was performed on a high-resolution orbital trap MS (Exactive, Thermo Fisher Scientific, Hemel Hempstead, UK) in ESI+/ESI– switching modes. MS spectra were acquired in a full scan mode (range: *m/z* 70–1400) with a resolution of 50,000. The capillary and heater temperatures were maintained at 275 °C and 150 °C, respectively. MS spray and capillary voltages were 4.5 kV (ESI+)/3.5 kV (ESI−) and 40 V (ESI+)/–30 V (ESI−), respectively. The flow rates (arbitrary unit) of the sheath, auxiliary, and sweep gas flow rates, for both modes, were 40, 5, and 1, respectively.

The extracts of the differentiated THP-1 cells treated with F-127- (*n* = 6), F-68- (*n* = 6), P-85- (*n* = 6), and PEG (*n* = 6)-coated PLGA NPs; plain PLGA NPs (*n* = 6); control (no treatment, *n* = 6); and reagent blank (*n* = 6) were randomized and analyzed in a single LC–MS run with the authentic standards. The column was conditioned for the analysis by injecting the QC (*n* = 6) at the beginning of the run; also, QC injections (*n* = 6) were interspaced in the analysis to check the performance of the LC–MS system for untargeted metabolomics.

### Data Analysis and Metabolite Identification

The acquired raw data sets from the LC–MS analysis of the extracts of untreated THP-1 cells (control) and the cells exposed to the different types of NPs and reagent blanks were processed using IDEOM as stated elsewhere.^34^ In brief, untargeted peak picking and peak matching were performed using XCMS^35^ and mzMatch,^36^ respectively. Noise filtering and putative metabolite identification were performed using IDEOM with the default parameters in which accurate mass and the retention times (RTs) of the authentic standards and the predicted RT (*p*RT) of the metabolite in the database were used for metabolite identification. Metabolites that were identified by matching with accurate mass and RT of the authentic standards were classified as level 1 identification according to the metabolomics standards initiative,^37,38^ whereas metabolites identified using accurate mass and *p*RT were considered as level 2 identification.

Multivariate analysis was performed using Simca P+13 (Umetrics, Umeå, Sweden) in which the data sets of the samples in the study were mean-centered, Pareto-scaled, and log-transformed. Principal component analysis (PCA) and orthogonal partial least squares-discriminant analysis (OPLS-DA) were performed to assess the performance of the analytical system and to find metabolic trends, clustering and separation in the metabolic profiles of the samples.^39^ The performance and robustness of the generated PCA and OPLS-DA were monitored using the fitness of model (*R*^2^*X*/*R*^2^*Y*) and predictive ability (*Q*^2^) values (leave-one-out (LOO) cross-validation).^40^ Variable importance in the projection (VIP) of the OPLS-DA and student *t*-test adjusted with a false discovery rate (adjusted *p*-value) using the Benjamini–Hochberg approach^41^ for the multiple testing problem were used to identify metabolites with a significant difference in the extracts of the differentiated THP-1 cells treated with F-127-, F-68-, P-85-, and PEG-coated PLGA NPs and PLGA NPs compared to untreated cells (control); VIP > 1.0 and adjusted *p*-value < 0.05 were considered significant.

The list of significantly altered metabolites were subjected to pathway analysis using MetaboAnalyst 4.0.^42^ Pathway analysis was performed to identify possible metabolic pathways affected due to the exposure of THP-1 cells to different NPs in the study. To avoid the probability of introducing false-positive identification into pathway enrichment analysis, preselected significant metabolites were only used.

## Results and Discussion

### Synthesis and Characterization of the PLGA Polymers

**Figure 1:**
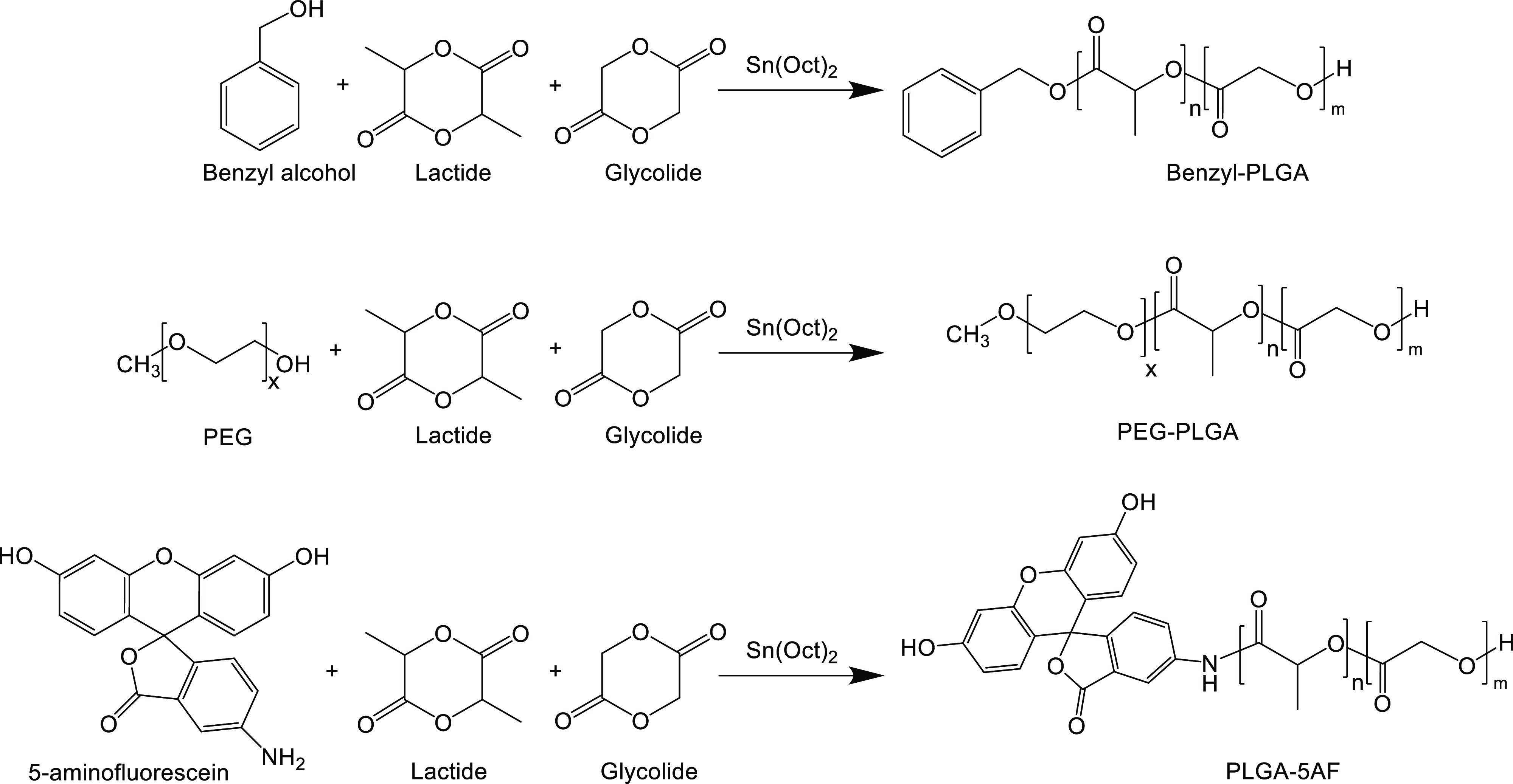
Schematic representation of the synthesis of PLGA, PLGA-5AF, and PEG–PLGA using ring-opening polymerization.

The synthesis of PLGA polymers is illustrated in Figure 1. All of the prepared polymers were synthesized by ring-opening polymerization of lactide and glycolide using benzyl alcohol, 5-aminoflourescin, and MPEO as initiators to produce PLGA, fluorescently labeled PLGA-5AF, and PEG–PLGA diblock copolymer, respectively. The purified polymer physical state was either a colorless powder for PLGA and PEG–PLGA or an orange powder for labeled PLGA-5AF. The final yields of PLGA (5.45 g) and PEG–PLGA (6 g) were 81 and 88%, respectively, whereas PLGA-5AF yield (3.3 g) was lower (50%) as expected due to the extensive washing cycles required to remove the traces of the free dye. The purity of the polymers was assessed using H^1^ NMR, and the obtained spectra (Figure S1) were in close accordance with previous studies,^43^ indicating that the prepared polymers were free from the residual monomer. Gel permeation chromatography was used to determine number average molecular masses (*M*_n_) of the prepared polymers compared to the polyester standard in which *M*_n_ values of PLGA, PEG–PLGA, and PLGA-5AF were found to be 9.9 × 10^3^, 1.1 × 10^4^, and 8.6 × 10^3^ g mol^–1^, respectively, and these results compare well to previously synthesized polymers with similar techniques.^44^ The polydispersity index for PEG–PLGA was 1.06; however, it was higher for PLGA and PLGA-5AF. This might be attributed to the fact that the hydroxyl and amino (anilinic) groups of benzyl alcohol and 5AF, respectively, are weak nucleophiles due to either close aromatic proximity or direct aromatic linkage, respectively, compared to the aliphatic hydroxyl group of PEG (Figure 1).

### Preparation and Characterization of the PLGA NPs

Plain and Pluronic-coated NPs were fabricated from PLGA and PLGA-5AF (4:1), while PEG–PLGA NPs were prepared from PEG–PLGA, PLGA, and PLGA-5AF (5:3:2). Only 20% of PLGA-5AF was required to prepare fluorescent NPs. Size and zeta potentials of the prepared NPs are given in Table S1. TEM showed that all the NPs in this study were spherical with smooth surfaces (Figure S2). All the obtained NPs had a similar size range of around 100 nm in diameter but varied zeta potentials (range: −22 to - 43 mv). The high negative zeta potential of the plain PLGA NPs (−43 ± 3 mv) may be attributed to the free carboxylic acid group at the end of the PLGA chains. However, the zeta potentials of the coated PLGA NPs (range: −22 to −36 mv) were significantly decreased compared to the plain PLGA NPs prepared by nanoprecipitation, ranging between −45 and 65 mv,^45^ and this, as expected, may be a result of the shielding effect of the nonionic coatings on the total negative surface charge of the NPs.^46^ This demonstrated that all the Pluronic(F-127, F-68, and P-85)-coated NPs were relatively uncharged compared to the plain PLGA NPs. However, F-127 coating resulted in the greatest change in zeta potential of the NPs, indicating that the higher molar mass of the F127 polymer, compared to F-68 and P-85, masked the surface charge of the NPs more efficiently.

### Cell Viability and the Cellular Uptake of the NPs by the Differentiated THP-1 Cells

**Figure 2:**
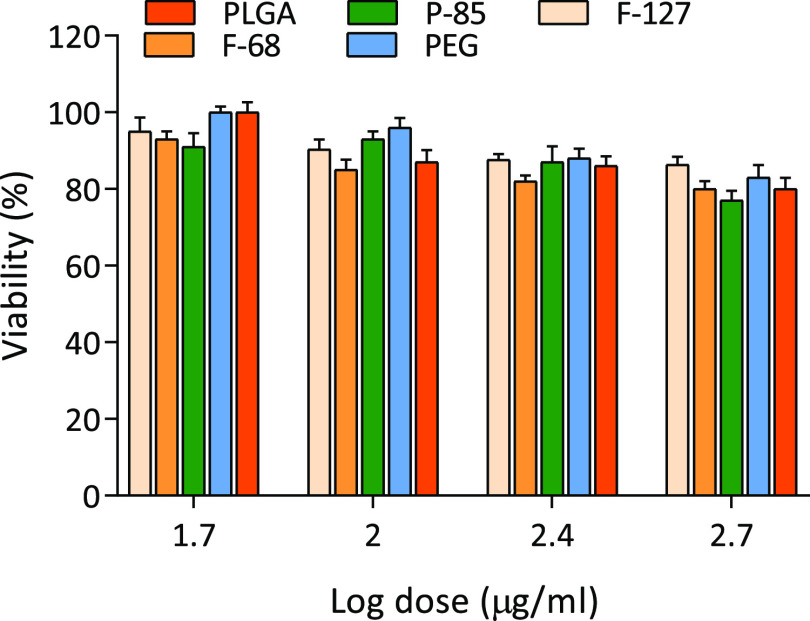
Cell viability of the differentiated THP-1 macrophages following exposure to plain, F-127-, F68-, and P-85-coated and PEGylated PLGA NPs. The cells were exposed to the different NPs for 24 h, and the cell viability was assessed using alamarBlue assay. The results are expressed as the average percentage of cell viability (*n* = 3).

**Figure 3:**
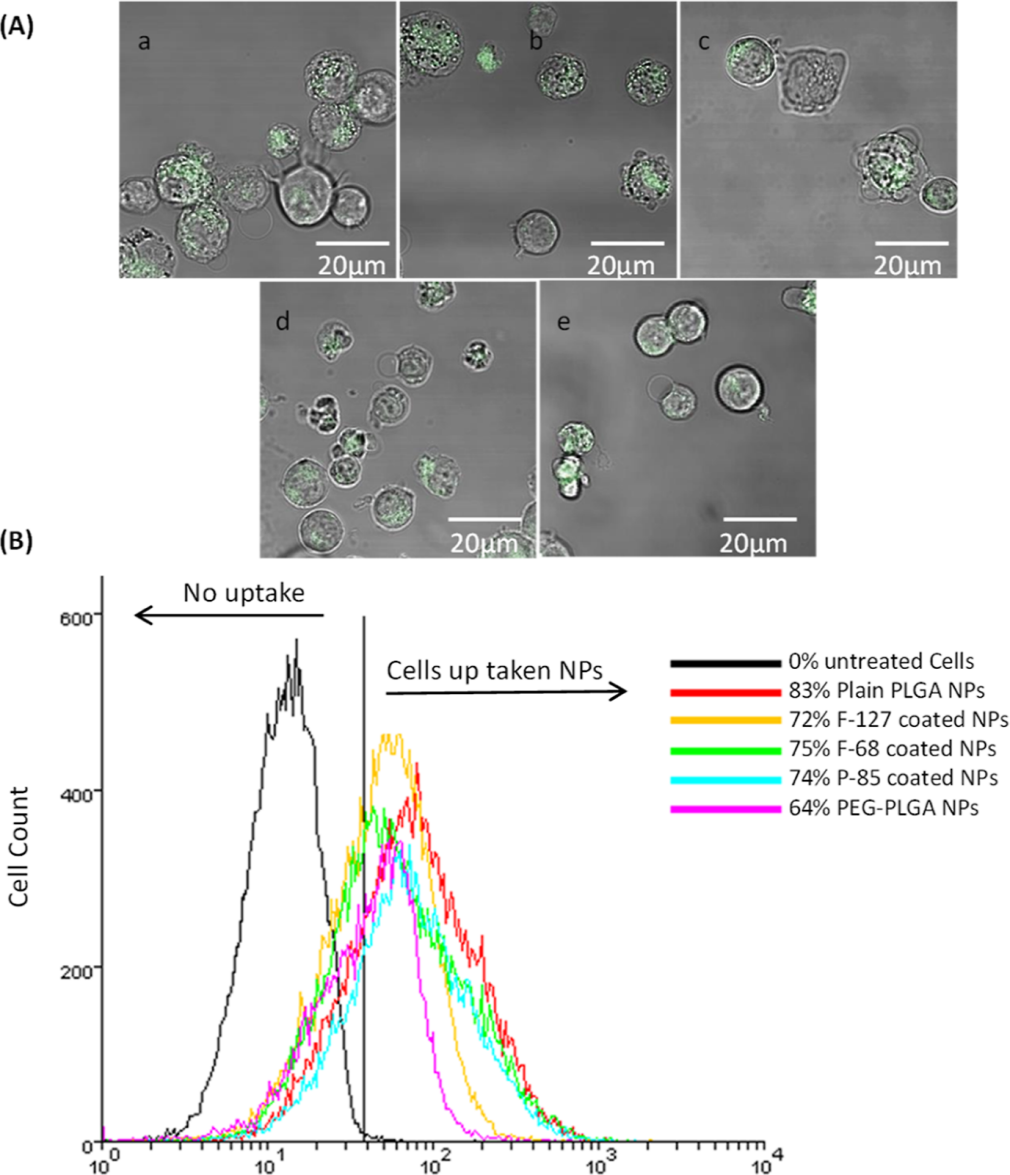
Cellular uptake of a: plain, b: F-127-coated, c: F-68-coated, d: P-85-coated, and e: PEGylated PLGA NPs by the differentiated THP-1 cells examined using confocal microscopy and flow cytometry. (A) Cellular uptake confocal microscopy images, the green color presents the NPs with different surface chemistries. (B) % cellular uptake of the NPs by the THP-1 cells examined using flow cytometry.

The differentiated THP-1 macrophage cells were grown in 24-well plates and exposed to different concentrations (50–500 μg/mL) of the synthesized NPs for 24 h. All the NPs were well-tolerated by the THP-1 cells at different concentrations up to 500 μg/mL evident with obtained results in which more than 75% of the exposed cells were viable (Figure 2). In terms of cellular uptake, confocal microscopy revealed that all the prepared NPs were readily taken up by the cells after 1 h exposure (Figure 3A). The rapid cellular uptake of NPs by THP-1 cells might be related to the fact that PMA enables the differentiation of THP-1 cells into phagocytic macrophages that subsequently engulf the NPs (the prepared NPs have a similar particle size and surface charge to viruses). Furthermore, flow cytometry analysis showed that all the PLGA NPs with the different surface chemistries were taken up by at least 64–83% of the cells after 1 h exposure (Figure 3B).

### Performance of the Analytical System for LC−MS Untargeted Metabolomics

The performance of the LC–MS system for untargeted metabolomics of the differentiated THP-1 cells treated with plain, Pluronics (F-127, F-68, and P-85), PEG-coated PLGA NPs, control (no treatment), and QC was assessed using relative standard deviations (RSDs) of all peaks present in the QC and PCA of all the detected peaks in the samples. The data sets of the analyzed samples in the study generated 8,039 peaks in which the RSDs were ≤ 30% for 73.4% of all the detected features in the QC (*n* = 6) and in accordance with the recommended threshold of variability for untargeted metabolomics.^47^ In this study, the PCA scores plot (Figure S3) was mainly used to check the performance of the analytical method using the QC samples and to observe any possible general trends. However, the poor clustering and separation of the sample groups in the PCA scores plot might be attributed to the fact that subtoxic concentrations of the tested materials were used on the cells to avoid the reduction in cell viability (>10–15%) which would have a significant unwanted effect on the metabolomics analysis (e.g., variations between the sample groups). In addition, THP-1 cell differentiation is a complex multistep process that might impart some within-group variations, leading to the poor clustering in the scores plot. Nevertheless, the QCs were adequately clustered in the middle of the PCA score plot and demonstrate that the instrument performance was satisfactory as suggested for metabolomics analysis.^48,49^ These univariate and multivariate analyses validate the analytical performance for LC-MS-based metabolic profiling.

### Data Analysis of the Metabolic Profiles of the Differentiated THP-1 Cells Exposed to NPs

**Figure 4:**
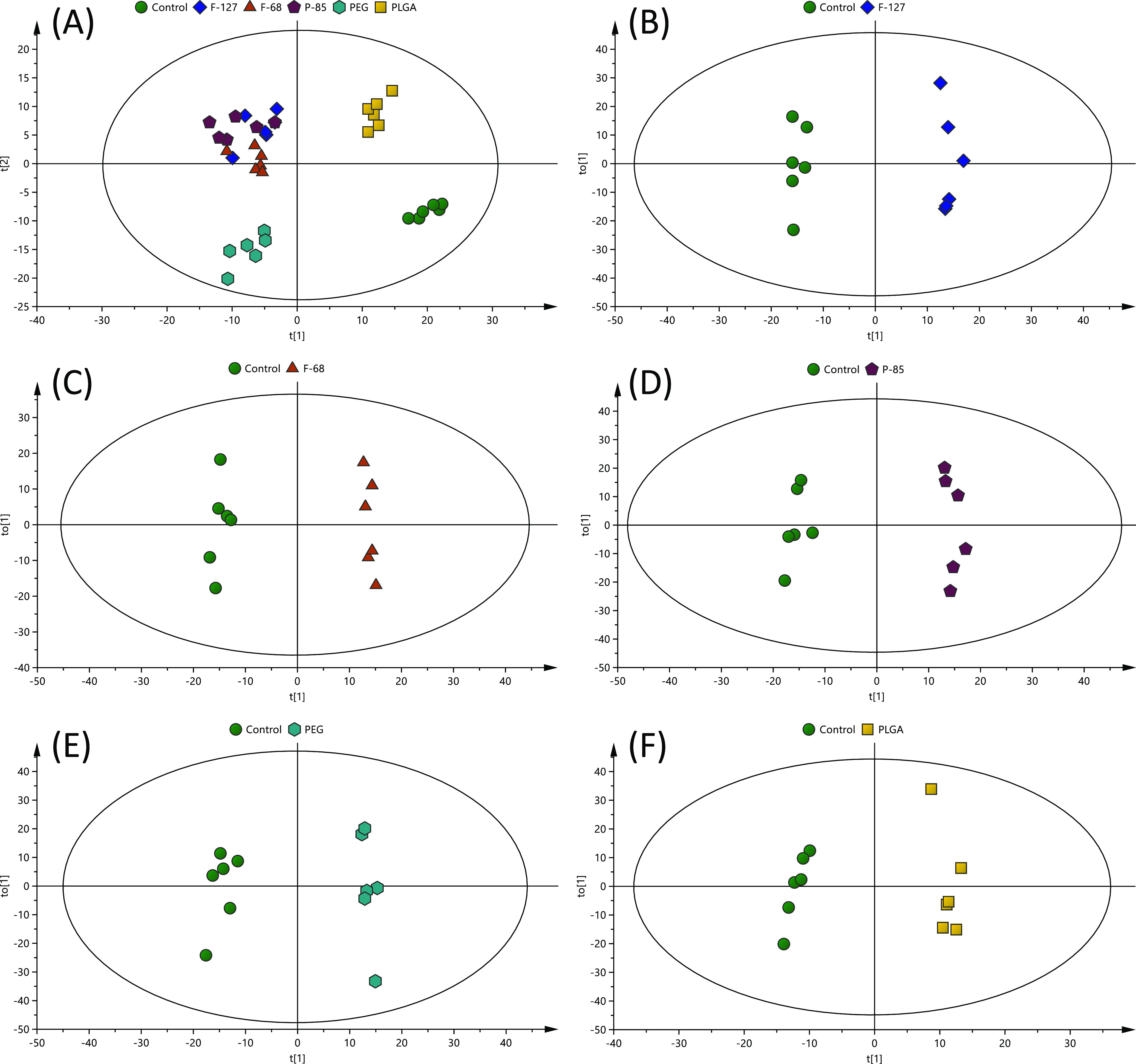
OPLS-DA scores plots of the metabolic profiles of the extracts of the differentiated THP-1 cells after 24 h treatment with different types of NPs (F-127, F-68, P-85, PEG, and PLGA) compared to the control (no treatment) analyzed with LC–MS. (A) OPLS-DA scores plot of all classes (*R*^2^*X* = 0.343, *R*^2^*Y*: 0.380, and *Q*^2^ = 0.295), and the rest are OPLS-DA scores plots of (B) F-127 (*R*^2^*X* = 0.422, *R*^2^*Y*: 0.992, and *Q*^2^ = 0.887), (C) F-68 (*R*^2^*X* = 0.332, *R*^2^*Y*: 0.993, and *Q*^2^ = 0.869), (D) P-85 (*R*^2^*X* = 0.438, *R*^2^*Y*: 0.989, and *Q*^2^ = 0.908), (E) PEG (*R*^2^*X* = 0.443, *R*^2^*Y*: 0.986, and *Q*^2^ = 0.867), and (F) PLGA (*R*^2^*X* = 0.357, *R*^2^*Y*: 0.984, and *Q*^2^ = 0.642) compared to the control (*n* = 6).

Multivariate analyses using PCA and OPLS-DA were performed on the data sets of the metabolic profiles of the differentiated THP-1 cells after 24 h exposure to plain PLGA, PLGA NPs coated with different surface functionalities (F-127, F-68, P-85, and PEG), and a control. No clustering and separation were observed between the metabolic profiles of the samples using PCA (Figure S3). Therefore, subsequent OPLS-DA was performed for modeling the differences between the different classes of the extracts; OPLS-DA (Figure 4A) (*R*^2^*X* = 0.343, *R*^2^*Y* = 0.380, and *Q*^2^ = 0.295) showed adequate separation and clustering of some classes in the samples, and therefore, it was used for further analysis.

The six biological replicates from each class of the samples were clustered closely in the OPLS-DA scores plot and separated from the control, indicating that within-class metabolic profiles were similar. However, the differentiated THP-1 cells exposed to plain PLGA NPs and PEG–PLGA NPs were separated clearly and clustered away from the Pluronic-coated PLGA NPs in the OPLS-DA scores plot (Figure 4A). Furthermore, the differentiated THP-1 cells exposed to Pluronic-coated PLGA NPs were closely clustered together with no separation observed in the OPLS-DA scores plot (Figure 4A). This cluster indicates that these types of NPs exerted similar effects on the cells, as expected, as these Pluronics NPs are structurally related and exhibited less negative surface charges compared to PEG–PLGA and plain PLGA NPs.

Further comparative OPLS-DA of each class of the differentiated THP-1 cells exposed to different NPs showed that they were clearly separated from the control (Figure 4B–F), indicating that their metabolic profiles are distinct from one class to another**.** Cross-validation of these OPLS-DA models was excellent with an *R*^2^*Y* of 0.984–0.993 and *Q*^2^ of 0.642–0.908; these values indicate that the models are robust as they are higher than the recommended values of 0.50 of *R*^2^*Y* and *Q*^2^ for untargeted metabolomics.^40^ These models, therefore, were used for the identification of significantly altered metabolites in the NP-exposed samples compared to the control.

### Metabolite Identification, Feature Selection, and Pathways in the Differentiated THP-1 Cells Related to the Exposure to NPs

**Figure 5:**
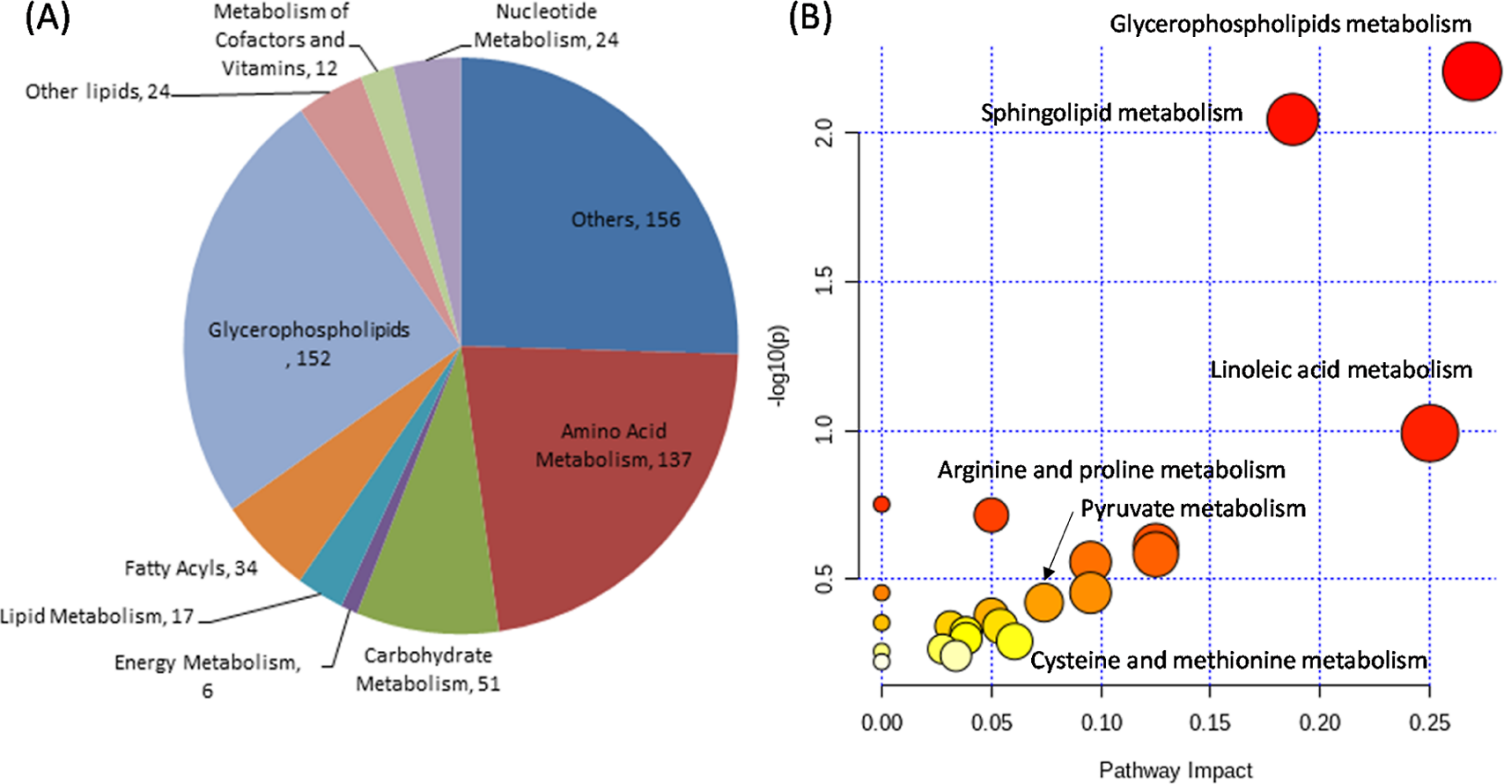
Overview of the metabolite classification and pathway analysis of the metabolites identified in the extracts of the differentiated THP-1 cells after 24 h treatment with different types of NPs (F-127, F-68, P-85, PEG, and PLGA) and control (no treatment) analyzed with LC–MS. (A) Classes of the identified metabolites and (B) pathway analysis of significantly altered metabolites between the different classes compared to the control.

**Figure 6:**
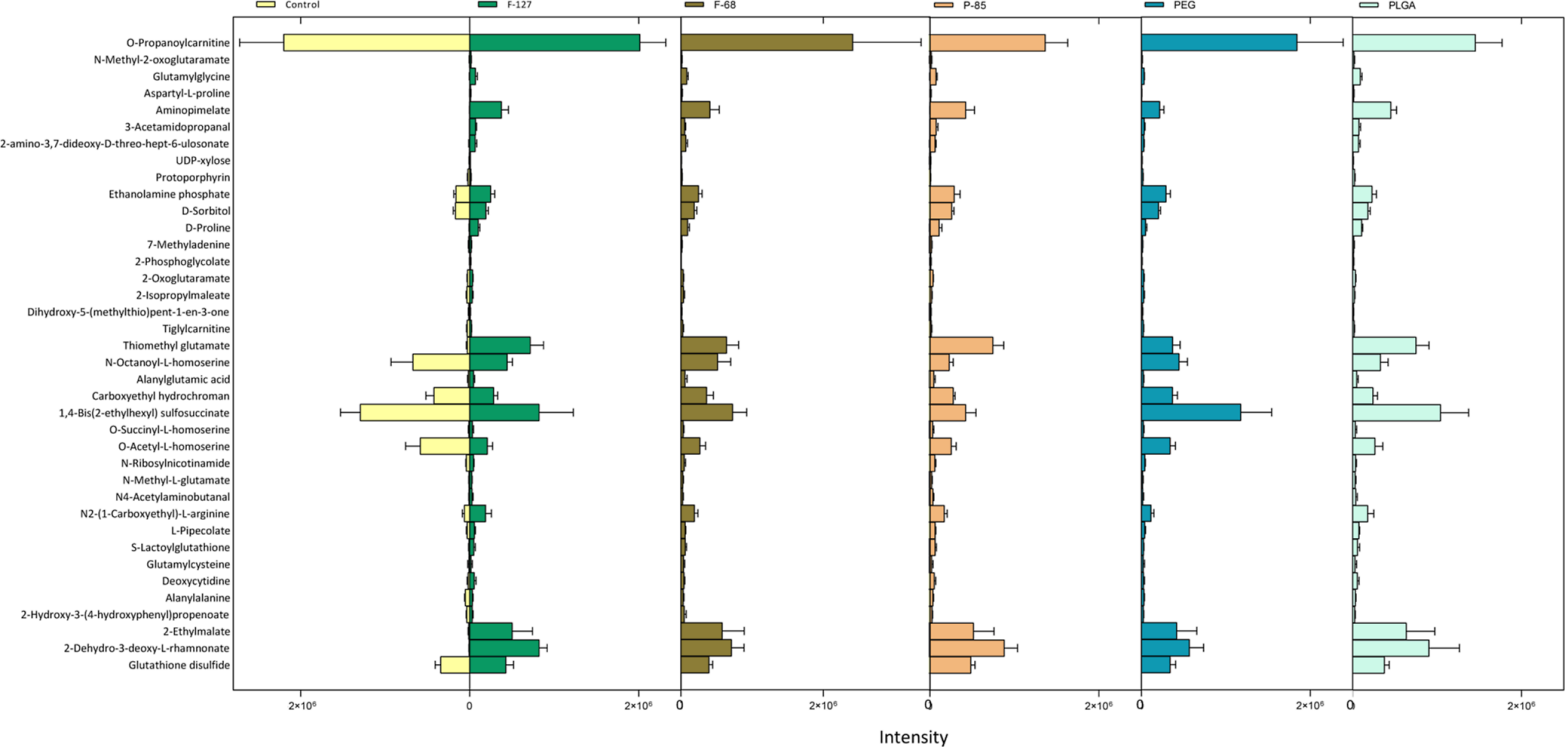
Comparison of the levels (peak intensities) of the significantly altered polar and semipolar metabolites detected in the extracts of the differentiated THP-1 cells treated with different types of NPs (F-127, F-68, P-85, PEG, and PLGA) compared to the control (no treatment) analyzed with LC–MS.

The detected metabolic features in the extracts of the differentiated THP-1 cells exposed to F-127-, F-68-, P-85-, and PEG-coated PLGA NPs, plain PLGA and control were subjected to metabolite identification using IDEOM. The accurate masses and RTs of these features were matched with those of the authentic standards or the *p*RT of the metabolites in the database. A total of 613 metabolites were putatively identified including essential and nonessential amino acids, glutathione-reduced and -oxidized forms, glycolysis, TCA cycle and urea cycle intermediate nucleotides, and different lipid classes including phosphatidylcholines (PC), phosphatidylethanolamines (PE), phosphatidylserines (PS), phosphatidylinositols (PI), and phosphatidylglycerols (PG) (Figure 5A). The significantly altered metabolites between each class of the samples in the study compared to the control were selected using VIP > 1.0 (OPLS-DA) and an adjusted *p*-value of < 0.05 (student *t*-test adjusted with the Benjamini–Hochberg approach^41^). As a result, 105 polar and semipolar metabolites and lipids were significantly altered in the NP-exposed samples compared to the control (full details are in Table S2). Table 1 shows the full list of the significantly altered metabolites, but not lipids, in each group of the samples compared to the control, whereas Figure 6 shows a mean of visual comparison of the intensities of these metabolites in each group of the samples in the study. Despite the use of 154 authentic standards to enhance the confidence in metabolite identification, most of the biologically relevant metabolites were of level 2 of confidence in identification, hence annotation, in which accurate mass and *p*RT were only used to perform identification.

**Table 1:** Significantly Altered Metabolites in the Extracts of the Differentiated THP-1 Macrophage Cells after Exposure to Different PLGA NPs Compared to the Control

					Log2 fold change: sample/control					adjusted *p*-value (sample/control)^b^					OPLS-DA VIP score (sample vs control)				
metabolite	accurate mass (Da)	mass error(ppm)	RT (min)	level of identification^a^	F-127	F-68	P-85	PEG	PLGA	F-127	F-68	P-85	PEG	PLGA	F-127	F-68	P-85	PEG	PLGA
glutathione disulfide	306.0760	0.15	11.07	accurate mass, RT (1)	0.3	0.2	0.5	0.0	0.1	4.0 × 10^–1^	5.8 × 10^–1^	1.3 × 10^–2^	1.0E+00	9.3 × 10^–1^	0.9	0.7	1.4	0.0	0.6
2-dehydro-3-deoxy-l-rhamnonate	162.0528	–0.09	5.39	accurate mass, *p*RT (2)	6.7	6.5	6.8	6.2	6.8	2.9 × 10^–7^	1.2 × 10^–4^	1.2 × 10^–5^	2.6 × 10^–4^	4.5 × 10^–3^	6.0	6.0	5.9	6.0	7.7
2-ethylmalate	162.0528	0.07	7.44	accurate mass, *p*RT (2)	5.3	5.5	5.3	5.0	5.6	7.0 × 10^–3^	1.5 × 10^–2^	5.8 × 10^–3^	2.2 × 10^–2^	2.9 × 10^–2^	5.3	5.5	5.1	5.2	6.8
2-hydroxy-3-(4-hydroxyphenyl)propenoate	180.0423	0.17	7.34	accurate mass, *p*RT (2)	–0.2	0.5	–0.4	–0.5	–0.4	7.6 × 10^–1^	7.0 × 10^–1^	1.0 × 10^–1^	1.5 × 10^–2^	5.5 × 10^–1^	0.6	0.8	1.3	1.6	1.5
alanylalanine	160.0848	–0.19	7.35	accurate mass, *p*RT (2)	–0.7	–0.7	–0.6	–0.8	–0.6	7.0 × 10^–3^	1.8 × 10^–2^	1.6 × 10^–2^	6.5 × 10^–4^	5.5 × 10^–3^	1.8	1.7	1.6	2.1	2.1
deoxycytidine	227.0907	0.18	5.78	accurate mass, *p*RT (2)	1.4	1.1	1.4	0.3	1.5	2.1 × 10^–2^	9.3 × 10^–3^	8.9 × 10^–3^	8.3 × 10^–1^	3.9 × 10^–2^	2.5	2.3	2.5	0.7	3.3
glutamylcysteine	250.0625	0.77	9.93	accurate mass, *p*RT (2)	0.7	1.8	1.1	1.1	1.4	8.0 × 10^–1^	5.0 × 10^–2^	5.0 × 10^–1^	5.3 × 10^–1^	6.0 × 10^–1^	0.6	1.5	1.0	0.6	1.5
*S*-lactoylglutathione	379.1050	0.27	8.80	accurate mass, *p*RT (2)	2.4	2.7	2.7	1.4	2.7	1.4 × 10^–3^	4.1 × 10^–4^	9.5 × 10^–5^	1.6 × 10^–3^	9.2 × 10^–3^	3.5	3.9	3.7	2.7	4.6
l-pipecolate	129.0790	–0.12	7.33	accurate mass, *p*RT (2)	0.9	1.0	1.0	0.4	1.3	3.8 × 10^–3^	3.2 × 10^–3^	1.1 × 10^–3^	2.8 × 10^–1^	1.7 × 10^–4^	2.1	2.2	2.2	1.2	3.3
N2-(1-carboxyethyl)-l-arginine	246.1330	0.71	10.52	accurate mass, *p*RT (2)	1.6	1.6	1.4	0.9	1.5	1.6 × 10^–2^	2.2 × 10^–3^	1.1 × 10^–3^	7.9 × 10^–2^	6.4 × 10^–2^	2.8	2.9	2.6	1.9	3.2
N4-acetylaminobutanal	129.0790	–0.01	5.41	accurate mass, *p*RT (2)	1.4	1.3	1.7	0.7	1.9	6.8 × 10^–2^	3.9 × 10^–3^	9.5 × 10^–3^	3.6 × 10^–1^	5.4 × 10^–2^	2.3	2.6	2.7	1.4	3.8
*N*-methyl-l-glutamate	161.0688	–0.20	10.35	accurate mass, *p*RT (2)	1.3	1.4	1.1	0.6	1.6	5.2 × 10^–5^	5.7 × 10^–3^	9.6 × 10^–3^	9.9 × 10^–2^	1.2 × 10^–3^	2.6	2.7	2.2	1.6	3.7
*N*-ribosylnicotinamide	254.0902	–0.27	18.21	accurate mass, *p*RT (2)	0.2	0.3	0.6	0.1	–0.1	2.7 × 10^–1^	8.1 × 10^–1^	2.6 × 10^–2^	8.6 × 10^–1^	9.8 × 10^–1^	0.9	0.5	1.5	0.4	0.6
N(Ω)-hydroxy-l-arginine	190.1066	–4.00	5.31	accurate mass, *p*RT (2)	6.4	6.1	6.5	5.2	6.5	1.62 × 10^–9^	1.34 × 10^–5^	6.48 × 10^–6^	5.28 × 10^–12^	2.55 × 10^–4^	5.9	5.9	5.7	5.6	7.5
*O*-acetyl-l-homoserine	161.0688	–0.29	8.84	accurate mass, *p*RT (2)	–1.5	–1.1	–1.2	–0.8	–1.1	7.8 × 10^–3^	2.6 × 10^–2^	1.3 × 10^–2^	7.7 × 10^–2^	6.1 × 10^–2^	2.7	2.3	2.3	1.8	2.8
*O*-succinyl-l-homoserine	219.0744	0.50	9.69	accurate mass, *p*RT (2)	1.5	1.6	1.6	1.1	1.6	7.1 × 10^–2^	4.8 × 10^–3^	8.9 × 10^–2^	7.6 × 10^–2^	1.7 × 10^–1^	2.2	2.5	2.0	1.6	2.8
1,4-Bis(2-ethylhexyl) sulfosuccinate	422.2339	0.26	4.01	accurate mass, *p*RT (2)	–0.7	–0.8	–1.6	–0.1	–0.3	2.1 × 10^–1^	1.4 × 10^–2^	3.1 × 10^–4^	9.4 × 10^–1^	7.9 × 10^–1^	1.6	2.0	2.8	0.5	1.2
carboxyethyl hydrochroman	278.1518	0.03	4.18	accurate mass, *p*RT (2)	–0.6	–0.2	–0.6	–0.2	–0.8	8.0 × 10^–2^	7.9 × 10^–1^	3.4 × 10^–2^	7.6 × 10^–1^	5.4 × 10^–2^	1.5	0.7	1.6	0.6	2.4
alanylglutamic acid	218.0903	–0.02	7.75	accurate mass, *p*RT (2)	1.6	1.8	1.6	0.6	1.7	5.1 × 10^–3^	4.5 × 10^–2^	7.5 × 10^–3^	4.3 × 10^–1^	1.7 × 10^–2^	2.8	3.0	2.7	1.5	3.6
*N*-octanoyl-l-homoserine	245.1627	0.03	7.45	accurate mass, *p*RT (2)	–0.6	–0.4	–1.5	–0.6	–1.0	3.2 × 10^–1^	7.5 × 10^–1^	1.8 × 10^–2^	3.7 × 10^–1^	1.9 × 10^–1^	1.3	0.9	2.6	1.3	2.6
thiomethyl glutamate	193.0410	0.51	7.32	accurate mass, *p*RT (2)	4.6	4.4	4.6	3.6	4.7	3.2 × 10^–5^	1.8 × 10^–4^	1.2 × 10^–5^	9.9 × 10^–5^	5.6 × 10^–5^	5.0	5.0	4.9	4.6	6.4
tiglylcarnitine	243.1470	–0.09	7.59	accurate mass, *p*RT (2)	–0.5	–0.1	–0.7	–0.4	–0.7	1.3 × 10^–1^	9.7 × 10^–1^	3.2 × 10^–2^	3.4 × 10^–1^	1.3 × 10^–1^	1.3	0.4	1.7	1.1	2.1
dihydroxy-5-(methylthio)pent-1-en-3-one	162.0350	–0.39	7.36	accurate mass, *p*RT (2)	–0.5	0.0	–0.1	0.0	–0.2	2.1 × 10^–2^	9.9 × 10^–1^	9.7 × 10^–1^	1.0E+00	8.2 × 10^–1^	1.5	0.1	0.3	0.1	0.9
2-isopropylmaleate	158.0579	–0.17	8.15	accurate mass, *p*RT (2)	–0.1	0.2	–0.6	–0.3	–0.6	9.1 × 10^–1^	8.5 × 10^–1^	3.9 × 10^–2^	6.5 × 10^–1^	1.7 × 10^–1^	0.3	0.5	1.5	0.9	1.9
2-oxoglutaramate	145.0375	–0.12	7.34	accurate mass, *p*RT (2)	0.5	0.4	0.5	0.1	0.5	6.2 × 10^–2^	1.3 × 10^–1^	2.3 × 10^–2^	9.5 × 10^–1^	9.2 × 10^–2^	1.4	1.2	1.4	0.3	1.8
2-phosphoglycolate	155.9823	–0.27	11.28	accurate mass, *p*RT (2)	2.6	2.0	2.6	1.9	2.6	2.5 × 10^–3^	6.3 × 10^–6^	8.9 × 10^–4^	1.6 × 10^–2^	5.4 × 10^–3^	3.7	3.4	3.5	3.1	4.6
7-methyladenine	149.0702	0.28	11.41	accurate mass, *p*RT (2)	0.6	0.2	0.6	0.1	0.2	1.1 × 10^–1^	8.5 × 10^–1^	3.8 × 10^–2^	9.2 × 10^–1^	9.5 × 10^–1^	1.5	0.6	1.6	0.5	0.6
d-proline	115.0633	0.17	7.33	accurate mass, *p*RT (2)	4.0	3.9	4.1	3.0	4.1	4.5 × 10^–6^	2.8 × 10^–4^	2.8 × 10^–4^	3.2 × 10^–4^	2.3 × 10^–6^	4.7	4.7	4.5	4.1	6.0
D-sorbitol	182.0791	0.17	10.32	accurate mass, *p*RT (2)	0.2	0.2	0.6	0.3	0.1	4.8 × 10^–1^	7.4 × 10^–1^	1.7 × 10^–3^	1.6 × 10^–1^	9.3 × 10^–1^	0.7	0.6	1.7	1.0	0.6
rthanolamine phosphate	141.0191	0.17	10.88	accurate mass, *p*RT (2)	0.6	0.6	0.8	0.8	0.5	1.8 × 10^–2^	3.5 × 10^–2^	1.5 × 10^–2^	4.7 × 10^–3^	1.8 × 10^–1^	1.7	1.7	1.9	2.1	1.8
protoporphyrin	562.2581	0.15	4.22	accurate mass, *p*RT (2)	–0.5	–0.4	–1.3	–0.2	0.1	1.2 × 10^–1^	2.9 × 10^–1^	1.4 × 10^–3^	6.8 × 10^–1^	9.9 × 10^–1^	1.4	1.1	2.4	0.6	0.4
UDP-xylose	536.0440	–0.87	10.71	accurate mass, *p*RT (2)	0.6	0.4	0.7	0.3	0.3	2.6 × 10^–4^	6.3 × 10^–3^	3.1 × 10^–3^	4.4 × 10^–1^	2.6 × 10^–1^	1.7	1.4	1.8	0.9	1.4
2-amino-3,7-dideoxy-D-*threo*-hept-6-ulosonate	191.0795	0.50	7.35	accurate mass, *p*RT (2)	3.7	3.8	3.7	2.5	3.9	2.9 × 10^–4^	4.3 × 10^–4^	3.3 × 10^–5^	2.4 × 10^–3^	2.5 × 10^–4^	3.5	3.7	3.4	2.6	4.6
3-acetamidopropanal	115.0633	0.12	5.38	accurate mass, *p*RT (2)	3.8	3.5	3.9	2.7	3.9	1.9 × 10^–6^	8.7 × 10^–5^	1.2 × 10^–4^	1.2 × 10^–3^	9.1 × 10^–5^	4.6	4.4	4.4	3.9	5.8
aminopimelate	175.0844	–0.17	7.32	accurate mass, *p*RT (2)	7.0	7.1	7.2	6.2	7.3	2.8 × 10^–5^	4.6 × 10^–4^	9.5 × 10^–5^	2.7 × 10^–5^	8.4 × 10^–6^	6.2	6.3	6.0	6.0	8.0
aspartyl-l-proline	230.0901	–0.60	7.40	accurate mass, *p*RT (2)	4.4	4.8	4.3	3.1	4.6	8.1 × 10^–4^	5.4 × 10^–3^	4.9 × 10^–3^	2.9 × 10^–2^	6.8 × 10^–4^	3.2	3.0	2.8	3.0	4.0
glutamylglycine	204.0747	0.49	8.58	accurate mass, *p*RT (2)	5.4	5.7	5.5	4.3	5.8	2.6 × 10^–4^	7.8 × 10^–5^	8.9 × 10^–6^	2.2 × 10^–6^	3.6 × 10^–5^	3.6	3.9	3.5	3.2	4.8
*N*-methyl-2-oxoglutaramate	159.0531	–0.18	5.37	accurate mass, *p*RT (2)	2.1	1.9	2.1	1.3	2.2	1.4 × 10^–4^	4.9 × 10^–3^	1.1 × 10^–3^	2.5 × 10^–2^	9.1 × 10^–3^	3.3	3.0	3.1	2.3	4.2
*O*-propanoylcarnitine	217.1313	–0.29	8.21	accurate mass, *p*RT (2)	–0.1	0.1	–0.7	–0.3	–0.6	8.3 × 10^–1^	9.6 × 10^–1^	4.4 × 10^–2^	7.8 × 10^–1^	2.1 × 10^–1^	0.4	0.2	1.6	0.8	1.9

a MSI protocol: Metabolomics Standard Initiative classification; level 1: the metabolites were identified using reference authentic standards by matching *m/z and* RT, level 2: putatively annotated metabolites using *m/z* and *p*RT of IDEOM library and no reference standards, level 3: putatively characterized metabolite classes, and level 4: unknowns.

b *p*-values were adjusted using Benjamini–Hochberg FDR.

The identified metabolites and lipids with a significant change in the extracts of the differentiated THP-1 cells exposed to NPs compared to the control were submitted for pathway analysis using MetaboAnalyst 4.0,^42^ and as a result, more than 20 metabolic pathways were found to be affected (Table S3). In these metabolic pathways, glycerophospholipid metabolism, sphingolipid metabolism, linoleic acid metabolism, biosynthesis of unsaturated fatty acids, arginine and proline metabolism, and alpha-linolenic acid metabolism were found to be highly altered (Figure 5B).

### Metabolic Signature of the NPs on the Differentiated THP-1 Cells

**Figure 7:**
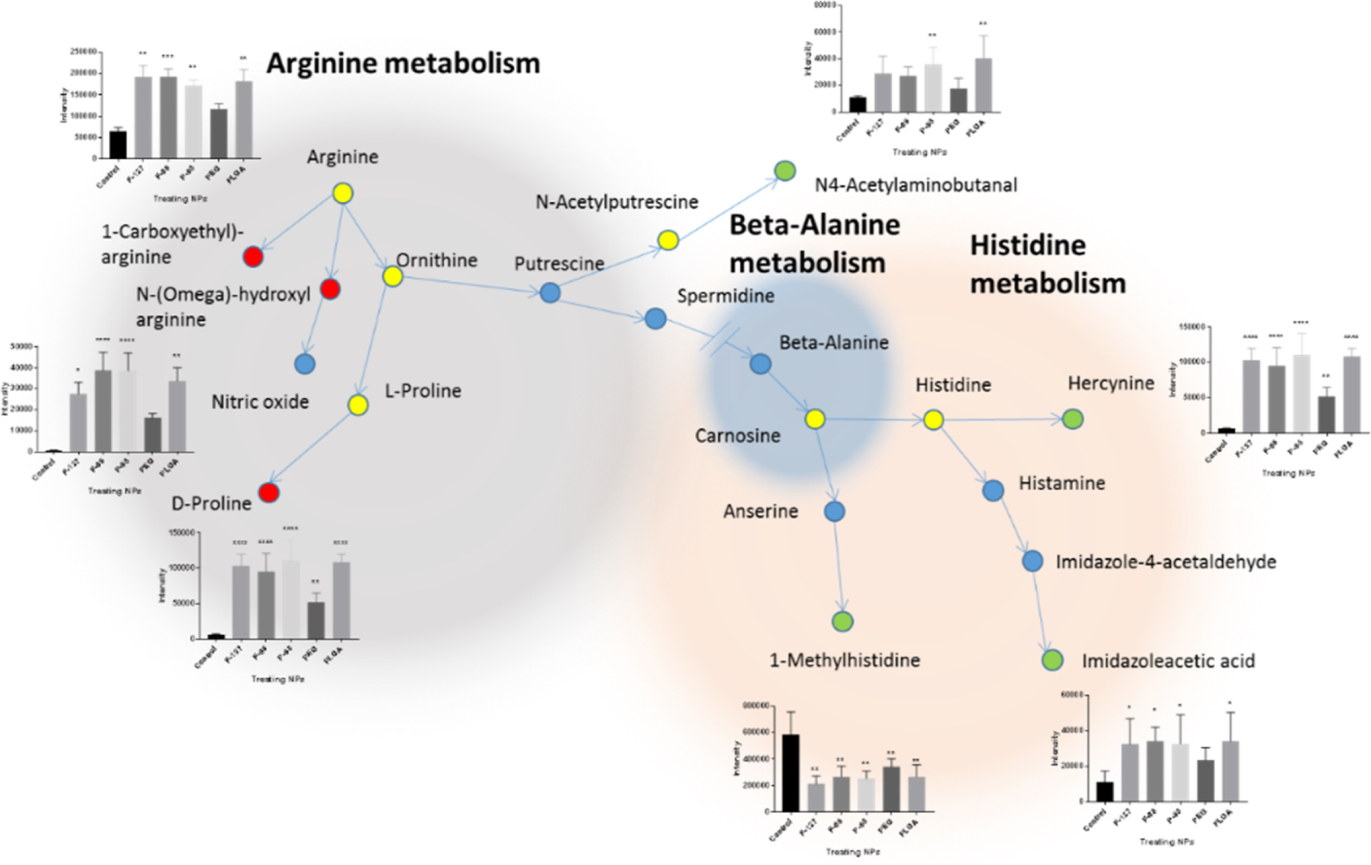
Metabolic pathway mapping of some of the identified metabolites in the extracts of the differentiated THP-1 cells treated with plain, Pluronic–coated, and PEG–PLGA NPs and control. Results are expressed as the average peak intensity ± SEM (****p < 0.0001, ***p < 0.001, **p < 0.01, and *p < 0.05).

A significant number of the perturbed metabolites were linked to amino acid metabolism, as shown in Figure 7. Amino acids are involved in many cellular metabolic pathways, providing a source of purine and pyrimidine which are required for nucleotide and nucleic acid synthesis and a carbon source after oxidation. Furthermore, some amino acids are precursors for other amino acid synthesis; for example, lysine, asparagine, methionine, and threonine are synthesized from aspartate. Glutamine, asparagine, proline, and arginine are synthesized from glutamate. Glycine and cysteine threonine are synthesized from serine, and tyrosine is synthesized by the hydroxylation of phenylalanine.^50^ Arginine and proline metabolic pathways in the differentiated THP-1 cells were found to be altered due to the treatment of the cells with NPs, in which levels of *N*-(Ω)-hydroxyarginine, N2-(D-1-carboxyethyl)-arginine, d-proline, and N4-acetylaminobutanal were significantly altered. Arginine is a semiessential amino acid with vital intracellular roles in addition to being a building block of proteins, and it is also involved in the urea cycle. Arginine is involved in detoxifying the ammonia that resulted from amino acid deamination by conversion into urea,^51^ as well as in the production of nitric oxide.^52^ Since *N*-(Ω)-hydroxyarginine is the first intermediate in NO synthesis through the oxidation of arginine, its accumulation may indicate an increased activity of nitric oxide synthase. A similar effect was seen upon treating macrophages with gold NPs but not PEG–gold NPs.^53^Figure 7 shows that PEG-coated PLGA NPs did not alter the levels of *N*-(Ω)-hydroxyarginine compared to the control as previously stated.^53^*N*-(Ω)-hydroxyarginine is known to inhibit arginase that catalyzes the final step in the urea cycle (Figure 7),^54^ suggesting that prolonged exposure and/or exposure to high concentrations of PLGA NPs may interfere with the urea cycle and subsequently accumulate toxic ammonia. Furthermore, the arginine metabolic pathway was affected in HepG2 human liver carcinoma cells when treated with graphene nanosheets,^18^ suggesting that such a metabolic effect is a result of nanomaterial physicochemical properties rather than surface chemistry. However, the chemistry of the surface cannot be ignored completely, as the plain PLGA NPs, which exhibited a similar surface charge to those reported for graphene nanosheets, had the most effect on arginine metabolism compared to the rest of NPs. There may also have been an important role of adsorbed media components in the cellular effects of the NPs, as the noncoated PLGA particles (as might also be expected for other relatively hydrophobic surface such as gold and graphene), likely were covered by a surface corona of amphiphilic biomolecules during cell culture. The results showing that PEG–PLGA NPs had the least impact on amino acid metabolism are in accordance with prior studies where PEGylation improves NPs biocompatibility,^55^ and again, this is known to be a consequence of reduced protein adsorption and foreign body response. However, while Pluronic-coated PLGA NPs should also display PEG chains from the surface to repel surface adsorption and therefore might be expected to be similarly inert as the PEG–PLGA NPs, the fact that the Pluronic-coated NPs were not inert is indicative of a different particle stability. In this case, the PEG chains of Pluronic-coated NPs were physically adsorbed and in equilibrium with desorbing Pluronic copolymer chains, whereas the PEG chains in the PEG PLGA NPs were chemically conjugated. Accordingly, while Pluronic copolymers may have desorbed readily over time from the PLGA particles intracellularly, the PEG chains in the PEG–PLGA particles were an integral part of kinetically trapped micellar-like NPs and unlikely to desorb at a rate which affected the macrophage cell lines in the timescales of our study.

The metabolite 3-acetaminopropanal (3-AAP) was found to be significantly altered in the differentiated THP-1 cells treated with NPs compared to the control. The production of 3-AAP is via a pathway including oxidation of *N*-acetylspermidine and *N*-acetylspermine; therefore, its accumulation along with other reactive aldehydes suggests a sign of oxidative stress.^56^ Furthermore, carboxyethyl hydrochroman is a major vitamin E metabolite, and it is a naturally occurring compound with antioxidant activity, for instance, when vitamin E or any of its metabolites undergo oxidation, they scavenge free radicals and stop oxidative damage in the process.^57^ Therefore, the decrease in carboxyethyl hydrochroman supports the hypothesis of an oxidative stress effect of NPs on THP-1 cells. These findings suggest that plain and Pluronic-coated PLGA NPs might induce oxidative stress on the differentiated THP-1 cells by enhancing the activity of NO synthase.

Glycerophospholipids are important components of the cell membrane.^58^ Exposure to Pluronic-coated and PEGylated NPs resulted in decreased levels of PC and PE, indicating that NPs cause alteration to the composition of the cell membrane and/or growth arrest.^59^ This is consistent with other studies in which macrophages were exposed to peptide-coated silver NPs^60^ and human keratinocytes to silver NPs^59^ and suggests that such an effect was related to the NP physiochemical properties and possible membrane disruption when a size range (i.e., in the nanoscale) is able to interact with the lipid components of the bilayer.

It has been previously reported that PLGA NPs at a concentration of 500 μg/mL altered glycolysis and TCA cycle,^61^ and this study showed that PLGA NPs with different surface modifications at clinically relevant concentration (100μg/mL) did not induce any significant changes in energy metabolism (glycolytic activities, TCA cycle, and fatty acid oxidation) or in nucleotide metabolism. This suggests that all the NPs with or without surface modification at clinically relevant concentrations were well tolerated by the cells, which aligns well with the cell viability assay results.

However, when taken together, the data suggest several important effects of the different NPs beyond those reported by simple metabolic activity assays and also highlight the roles that surface modifications and specific amphiphile chemistries can have at the subcellular level in macrophages. It has long been established that amphiphilic triblock copolymers of the Pluronics type (and other classes, such as Synperonics) form micelles in solution, and this has been exploited extensively for use in pharmaceutical applications, where the polymers are included in formulations as drug solubility enhancers. It has also been shown that, dependent on their chemical composition, certain PEO-PPO-PEO copolymers can mediate either adverse or beneficial cellular responses such as cytotoxicity, ATP and glutathione depletion, reversal of multidrug resistance in tumor cells, or inhibition of *P*-glycoprotein.^62^ Thus, while PEO-PPO-PEO copolymers have been largely considered as inert and safe excipients, some of them elicit marked biological responses, both in vitro and in vivo,^62^ and it is perhaps not surprising that we observed changes in metabolic pathways in cells exposed to Pluronic-coated PLGA NPs. It has been previously demonstrated that adverse cellular effects caused by PEO-PPO-PEO copolymers were dependent on their chemical structure.^8,63^ In addition these prior studies indicated a strong correlation of the Pluronic amphiphile structure with their affinity for biological membranes, and thus, the alterations in glycerophospholipids are in accordance with a membrane disruption and induced oxidative stress mechanism. It is likely that the greater effects of the Pluronic-coated NPs compared to the PEG–PLGA MPs were due to desorption of the Pluronics from the PLGA surface and subsequent interaction of free triblock polymer chains with the membrane and subcellular components in a manner not possible for PEG–PLGANPs. Similar desorption of PEG–PLGA chains from the kinetically trapped micellar-like PEG–PLGA NPs would have taken place at a much lower rate, leading to fewer pronounced membrane disruption effects over the time periods of our assays.

## Conclusions

In this study, a cell-based metabolomics approach was applied to investigate the biological impact of PLGA NPs with different surface functionalities on THP-1-derived macrophages using LC–MS. The metabolomics method revealed that plain PLGA NPs and surface-modified ones were well tolerated by the cells tested; nevertheless, there were several metabolic changes that are comparable to those caused by some metal oxide NPs and other nanomaterials. Exposure to plain and Pluronic-coated PLGA NPs led to alterations in amino acid metabolism, which could interfere with the urea cycle and induce signs of oxidative stress. Pluronic-coated and PEG–PLGA NPs also altered some glycerophospholipids, which are core membrane components, and such effects could lead to growth arrest and membrane integrity alterations. PEG–PLGA NPs had the least impact on the cellular metabolism in terms of metabolite fold changes, and these data agreed well with prior reports that a persistent and nondesorbing PEG layer improves the cytocompatibility of nanoparticulates. Finally, our data highlight the importance of understanding the potential impact of NPs and their different coatings on functional properties of macrophages regardless of their payload. This needs to be considered in the clinical application of NPs particularly where macrophages are the therapeutic targets. The changes in metabolic pathways altered and the potential for induction of oxidative stress may also be of clinical importance in long-term repeated dosing with surfactant-coated NPs.

## Supporting Information Available

Composition of the authentic standard mixtures coanalyzed with the samples using LC-HRMS for metabolite identification; significantly altered metabolites in the extract of activated THP-1 cells due to exposure of NPs with different coating materials; and affected metabolic pathways in the activated THP-1 cells due to exposure of NPs with different coating materials (XLSX)
Characterization of the prepared NPs based on size measurement and zeta potential determined using Malvern Zetasizer; 1H NMR spectra of (A) PLGA, (B) PEG–PLGA, and (C) PLGA-5AF in CDCl3; morphology of the prepared NPs examined using TEM; and PCA score plots of the metabolic profiles of the extract of the differentiated THP-1 cells after 24 h treatment with different types of NPs compared to the control (no treatment) (PDF)

## References

[ref1] Lee S. W.; Kim Y. M.; Cho C. H.; Kim Y. T.; Kim S. M.; Hur S. Y.; Kim J. H.; Kim B. G.; Kim S. C.; Ryu H. S.; Kang S. B. An Open-Label, Randomized, Parallel, Phase II Trial to Evaluate the Efficacy and Safety of a Cremophor-Free Polymeric Micelle Formulation of Paclitaxel as First-Line Treatment for Ovarian Cancer: A Korean Gynecologic Oncology Group Study (KGOG-3021). Cancer Res. Treat. 2018, 50, 195–203. 10.4143/crt.2016.376. 28324920 PMC5784626

[ref2] Conejos-Sánchez I.; Cardoso I.; Saraiva M. J.; Vicent M. J. Targeting a rare amyloidotic disease through rationally designed polymer conjugates. J. Controlled Release 2014, 178, 95–100. 10.1016/j.jconrel.2014.01.019. 24486260

[ref3] Shen X.; Li T.; Xie X.; Feng Y.; Chen Z.; Yang H.; Wu C.; Deng S.; Liu Y. PLGA-Based Drug Delivery Systems for Remotely Triggered Cancer Therapeutic and Diagnostic Applications. Front. Bioeng. Biotechnol. 2020, 8, 381 10.3389/fbioe.2020.00381. 32432092 PMC7214837

[ref4] Stolnik S.; Dunn S. E.; Garnett M. C.; Davies M. C.; Coombes A. G. A.; Taylor D. C.; Irving M. P.; Purkiss S. C.; Tadros T. F.; Davis S. S.; Illum L. Surface Modification Of Poly(Lactide-Co-Glycolide) Nanospheres By Biodegradable Poly(Lactide)-Poly(Ethylene Glycol) Copolymers. Pharm. Res. 1994, 11, 1800–1808. 10.1023/a:1018931820564. 7899246

[ref5] Monteiro P. F.; Travanut A.; Conte C.; Alexander C. Reduction-responsive polymers for drug delivery in cancer therapy-Is there anything new to discover?. Nanomed. Nanobiotechnol. 2021, 13, e1678 10.1002/wnan.1678. 33155421

[ref6] Werner M. E.; Cummings N. D.; Sethi M.; Wang E. C.; Sukumar R.; Moore D. T.; Wang A. Z. Preclinical evaluation of Genexol-PM, a nanoparticle formulation of paclitaxel, as a novel radiosensitizer for the treatment of non-small cell lung cancer. Int. J. Radiat. Oncol., Biol., Phys. 2013, 86, 463–468. 10.1016/j.ijrobp.2013.02.009. 23708084 PMC3750707

[ref7] Armstrong J. K.; Hempel G.; Koling S.; Chan L. S.; Fisher T.; Meiselman H. J.; Garratty G. Antibody against poly(ethylene glycol) adversely affects PEG-asparaginase therapy in acute lymphoblastic leukemia patients. Cancer 2007, 110, 103–111. 10.1002/cncr.22739. 17516438

[ref8] Redhead M.; Mantovani G.; Nawaz S.; Carbone P.; Gorecki D. C.; Alexander C.; Bosquillon C. Relationship between the Affinity of PEO-PPO-PEO Block Copolymers for Biological Membranes and Their Cellular Effects. Pharm. Res. 2012, 29, 1908–1918. 10.1007/s11095-012-0716-6. 22392332

[ref9] Sivaram A. J.; Wardiana A.; Alcantara S.; Sonderegger S. E.; Fletcher N. L.; Houston Z. H.; Howard C. B.; Mahler S. M.; Alexander C.; Kent S. J.; Bell C. A.; Thurecht K. J. Controlling the Biological Fate of Micellar Nanoparticles: Balancing Stealth and Targeting. ACS Nano 2020, 14, 13739–13753. 10.1021/acsnano.0c06033. 32936613

[ref10] Song D.; Cui J.; Ju Y.; Faria M.; Sun H.; Howard C. B.; Thurecht K. J.; Caruso F. Cellular Targeting of Bispecific Antibody-Functionalized Poly(ethylene glycol) Capsules: Do Shape and Size Matter?. ACS Appl. Mater. Interfaces 2019, 11, 28720–28731. 10.1021/acsami.9b10304. 31369234

[ref11] Alakhova D. Y.; Rapoport N. Y.; Batrakova E. V.; Timoshin A. A.; Li S.; Nicholls D.; Alakhov V. Y.; Kabanov A. V. Differential metabolic responses to pluronic in MDR and non-MDR cells: A novel pathway for chemosensitization of drug resistant cancers. J. Controlled Release 2010, 142, 89–100. 10.1016/j.jconrel.2009.09.026. PMC3113470 19815037

[ref12] Anreddy R. N.; Yellu N. R.; Devarakonda K. R. Oxidative biomarkers to assess the nanoparticle-induced oxidative stress. Methods Mol. Biol. 2013, 1028, 205–219. 10.1007/978-1-62703-475-3_13. 23740122

[ref13] Siddiqui M. A.; Alhadlaq H. A.; Ahmad J.; Al-Khedhairy A. A.; Musarrat J.; Ahamed M. Copper oxide nanoparticles induced mitochondria mediated apoptosis in human hepatocarcinoma cells. PLoS One 2013, 8, e69534 10.1371/journal.pone.0069534. 23940521 PMC3734287

[ref14] Trouiller B.; Reliene R.; Westbrook A.; Solaimani P.; Schiestl R. H. Titanium dioxide nanoparticles induce DNA damage and genetic instability in vivo in mice. Cancer Res 2009, 69, 8784 10.1158/0008-5472.CAN-09-2496. 19887611 PMC3873219

[ref15] Gioria S.; Lobo Vicente J.; Barboro P.; La Spina R.; Tomasi G.; Urbán P.; Kinsner-Ovaskainen A.; François R.; Chassaigne H. A combined proteomics and metabolomics approach to assess the effects of gold nanoparticlesin vitro. Nanotoxicology 2016, 10, 736–748. 10.3109/17435390.2015.1121412. 26647645 PMC4898143

[ref16] Hirsch C.; Roesslein M.; Krug H. F.; Wick P. Nanomaterial cell interactions: are current in vitro tests reliable?. Nanomedicine 2011, 6, 837–847. 10.2217/nnm.11.88. 21793675

[ref17] Kong B.; Seog J. H.; Graham L. M.; Lee S. B. Experimental considerations on the cytotoxicity of nanoparticles. Nanomedicine 2011, 6, 929–941. 10.2217/nnm.11.77. 21793681 PMC3196306

[ref18] Jiao G.; Li X.; Zhang N.; Qiu J.; Xu H.; Liu S. Metabolomics study on the cytotoxicity of graphene. RSC Adv. 2014, 4, 44712–44717. 10.1039/c4ra06312k.

[ref19] Fiehn O. Metabolomics - the link between genotypes and phenotypes. Plant Mol. Biol. 2002, 48, 155–171. 10.1007/978-94-010-0448-0_11. 11860207

[ref20] Xiao J. F.; Zhou B.; Ressom H. W. Metabolite identification and quantitation in LC-MS/MS-based metabolomics. TrAC, Trends Anal. Chem. 2012, 32, 1–14. 10.1016/j.trac.2011.08.009. PMC3278153 22345829

[ref21] Patti G. J.; Yanes O.; Siuzdak G. Metabolomics: the apogee of the omics trilogy. Nat. Rev. Mol. Cell Biol. 2012, 13, 263–269. 10.1038/nrm3314. 22436749 PMC3682684

[ref22] Alazzo A.; Al-Natour M. A.; Spriggs K.; Stolnik S.; Ghaemmaghami A.; Kim D.-H.; Alexander C. Investigating the intracellular effects of hyperbranched polycation-DNA complexes on lung cancer cells using LC-MS-based metabolite profiling. Mol. Omics 2019, 15, 77–87. 10.1039/c8mo00139a. 30706066

[ref23] Dahabiyeh L. A.; Mahmoud N. N.; Al-Natour M. A.; Safo L.; Kim D.-H.; Khalil E. A.; Abu-Dahab R. Phospholipid-Gold Nanorods Induce Energy Crisis in MCF-7 Cells: Cytotoxicity Evaluation Using LC-MS-Based Metabolomics Approach. Biomolecules 2021, 11, 364 10.3390/biom11030364. 33673519 PMC7997200

[ref24] Al-Natour M. A.; Alazzo A.; Ghaemmaghami A. M.; Kim D.-H.; Alexander C. LC-MS metabolomics comparisons of cancer cell and macrophage responses to methotrexate and polymer-encapsulated methotrexate. Int. J. Pharm.: X 2019, 1, 100036 10.1016/j.ijpx.2019.100036. 31993584 PMC6977166

[ref25] Johnston H. J.; Hutchison G.; Christensen F. M.; Peters S.; Hankin S.; Stone V. A review of the in vivo and in vitro toxicity of silver and gold particulates: particle attributes and biological mechanisms responsible for the observed toxicity. Crit. Rev. Toxicol. 2010, 40, 328–346. 10.3109/10408440903453074. 20128631

[ref26] Donaldson K.; Murphy F. A.; Duffin R.; Poland C. A. Asbestos, carbon nanotubes and the pleural mesothelium: a review of the hypothesis regarding the role of long fibre retention in the parietal pleura, inflammation and mesothelioma. Part. Fibre Toxicol. 2010, 7, 1–17. 10.1186/1743-8977-7-5. 20307263 PMC2857820

[ref27] Grabowski N.; Hillaireau H.; Vergnaud J.; Santiago L. A.; Kerdine-Romer S.; Pallardy M.; Tsapis N.; Fattal E. Toxicity of surface-modified PLGA nanoparticles toward lung alveolar epithelial cells. Int. J. Pharm. 2013, 454, 686–694. 10.1016/j.ijpharm.2013.05.025. 23747506

[ref28] Chanput W.; Mes J. J.; Wichers H. J. THP-1 cell line: an in vitro cell model for immune modulation approach. Int. Immunopharmacol. 2014, 23, 37–45. 10.1016/j.intimp.2014.08.002. 25130606

[ref29] Abuawad A.; Mbadugha C.; Ghaemmaghami A. M.; Kim D.-H. Metabolic characterisation of THP-1 macrophage polarisation using LC-MS-based metabolite profiling. Metabolomics 2020, 16, 33 10.1007/s11306-020-01656-4. 32114632 PMC7049298

[ref30] Fessi H.; Puisieux F.; Devissaguet J. P.; Ammoury N.; Benita S. Nanocapsule formation by interfacial polymer deposition following solvent displacement. Int. J. Pharm. 1989, 55, R1–R4. 10.1016/0378-5173(89)90281-0.

[ref31] Park E. K.; Jung H. S.; Yang H. I.; Yoo M. C.; Kim C.; Kim K. S. Optimized THP-1 differentiation is required for the detection of responses to weak stimuli. Inflammation Res. 2007, 56, 45–50. 10.1007/s00011-007-6115-5. 17334670

[ref32] Creek D. J.; Jankevics A.; Breitling R.; Watson D. G.; Barrett M. P.; Burgess K. E. Toward global metabolomics analysis with hydrophilic interaction liquid chromatography-mass spectrometry: improved metabolite identification by retention time prediction. Anal. Chem. 2011, 83, 8703–8710. 10.1021/ac2021823. 21928819

[ref33] Surrati A.; Linforth R.; Fisk I. D.; Sottile V.; Kim D. H. Non-destructive characterisation of mesenchymal stem cell differentiation using LC-MS-based metabolite footprinting. Analyst 2016, 141, 3776–3787. 10.1039/c6an00170j. 27102615

[ref34] Creek D. J.; Jankevics A.; Burgess K. E.; Breitling R.; Barrett M. P. IDEOM: an Excel interface for analysis of LC-MS-based metabolomics data. Bioinformatics 2012, 28, 1048–1049. 10.1093/bioinformatics/bts069. 22308147

[ref35] Tautenhahn R.; Böttcher C.; Neumann S. Highly sensitive feature detection for high resolution LC/MS. BMC Bioinf. 2008, 9, 504 10.1186/1471-2105-9-504. PMC2639432 19040729

[ref36] Scheltema R. A.; Jankevics A.; Jansen R. C.; Swertz M. A.; Breitling R. PeakML/mzMatch: a file format, Java library, R library, and tool-chain for mass spectrometry data analysis. Anal. Chem. 2011, 83, 2786–2793. 10.1021/ac2000994. 21401061

[ref37] Sumner L. W.; Amberg A.; Barrett D.; Beale M. H.; Beger R.; Daykin C. A.; Fan T. W. M.; Fiehn O.; Goodacre R.; Griffin J. L.; Hankemeier T.; Hardy N.; Harnly J.; Higashi R.; Kopka J.; Lane A. N.; Lindon J. C.; Marriott P.; Nicholls A. W.; Reily M. D.; Thaden J. J.; Viant M. R. Proposed minimum reporting standards for chemical analysis. Metabolomics 2007, 3, 211–221. 10.1007/s11306-007-0082-2. 24039616 PMC3772505

[ref38] Sumner L. W.; Lei Z.; Nikolau B. J.; Saito K.; Roessner U.; Trengove R. Proposed quantitative and alphanumeric metabolite identification metrics. Metabolomics 2014, 10, 1047 10.1007/s11306-014-0739-6.

[ref39] Boccard J.; Rutledge D. N. A consensus orthogonal partial least squares discriminant analysis (OPLS-DA) strategy for multiblock Omics data fusion. Anal. Chim. Acta 2013, 769, 30–39. 10.1016/j.aca.2013.01.022. 23498118

[ref40] Eriksson L.; Johansson E.; Kettaneh-Wold N. J.; Trygg C. W.; Wold S. Multi- and Megavariate Data Analysis: Basic Principles and Applications, 2nd ed.; Umetrics AB: Umea, 2006; Vol. Part I.

[ref41] Benjamini Y.; Hochberg Y. Controlling the False Discovery Rate: A Practical and Powerful Approach to Multiple Testing. J. R. Stat. Soc. Series B Stat. Methodol. 1995, 57, 289–300. 10.1111/j.2517-6161.1995.tb02031.x.

[ref42] Chong J.; Wishart D. S.; Xia J. Using MetaboAnalyst 4.0 for Comprehensive and Integrative Metabolomics Data Analysis. Curr. Protoc. Bioinf. 2019, 68, e86 10.1002/cpbi.86. 31756036

[ref43] Erbetta C. D. A. C.; Alves R. J.; Resende J. M. e.; Freitas R. F. d. S.; Sousa R. G. d. Synthesis and Characterization of Poly(D,L-Lactide-co-Glycolide) Copolymer. J. Biomater. Nanobiotechnol. 2012, 3, 208–225.

[ref44] Al-Natour M. A.; Yousif M. D.; Cavanagh R.; Abouselo A.; Apebende E. A.; Ghaemmaghami A.; Kim D.-H.; Aylott J. W.; Taresco V.; Chauhan V. M.; Alexander C. Facile Dye-Initiated Polymerization of Lactide-Glycolide Generates Highly Fluorescent Poly(lactic-co-glycolic Acid) for Enhanced Characterization of Cellular Delivery. ACS Macro Lett. 2020, 9, 431–437. 10.1021/acsmacrolett.9b01014. 35648548

[ref45] Stromberg Z. R.; Lisa Phipps M.; Magurudeniya H. D.; Pedersen C. A.; Rajale T.; Sheehan C. J.; Courtney S. J.; Bradfute S. B.; Hraber P.; Rush M. N.; Kubicek-Sutherland J. Z.; Martinez J. S. Formulation of stabilizer-free, nontoxic PLGA and elastin-PLGA nanoparticle delivery systems. Int. J. Pharm. 2021, 597, 120340 10.1016/j.ijpharm.2021.120340. 33545284

[ref46] Barnes T. J.; Prestidge C. A. PEO–PPO–PEO Block Copolymers at the Emulsion Droplet–Water Interface. Langmuir 2000, 16, 4116–4121. 10.1021/la991217d.

[ref47] Begou O.; Gika H. G.; Theodoridis G. A.; Wilson I. D. Quality Control and Validation Issues in LC-MS Metabolomics. In Metabolic Profiling: Methods and Protocols, Theodoridis G. A., Gika H. G., Wilson I. D., Eds.; Springer: New York, NY, 2018; pp 15–26. 10.1007/978-1-4939-7643-0_2 29654580

[ref48] Want E. J.; Wilson I. D.; Gika H.; Theodoridis G.; Plumb R. S.; Shockcor J.; Holmes E.; Nicholson J. K. Global metabolic profiling procedures for urine using UPLC-MS. Nat. Protoc. 2010, 5, 1005–1018. 10.1038/nprot.2010.50. 20448546

[ref49] Gika H. G.; Theodoridis G. A.; Wingate J. E.; Wilson I. D. Within-Day Reproducibility of an HPLC–MS-Based Method for Metabonomic Analysis: Application to Human Urine. J. Proteome Res. 2007, 6, 3291–3303. 10.1021/pr070183p. 17625818

[ref50] Wu G. Amino acids: metabolism, functions, and nutrition. Amino Acids 2009, 37, 1–17. 10.1007/s00726-009-0269-0. 19301095

[ref51] Visek W. J. Arginine needs, physiological state and usual diets. A reevaluation. J. Nutr. 1986, 116, 36–46. 10.1093/jn/116.1.36. 3080558

[ref52] Tapiero H.; Mathé G.; Couvreur P.; Tew K. D. I. Arginine. Biomed. Pharmacother. 2002, 56, 439–445. 10.1016/s0753-3322(02)00284-6. 12481980

[ref53] Du L.; Miao X.; Jia H.; Gao Y.; Liu K.; Zhang X.; Liu Y. Detection of nitric oxide in macrophage cells for the assessment of the cytotoxicity of gold nanoparticles. Talanta 2012, 101, 11–16. 10.1016/j.talanta.2012.08.044. 23158284

[ref54] Morris Jr S. M. Jr Recent advances in arginine metabolism: roles and regulation of the arginases. Br. J. Pharmacol. 2009, 157, 922–930. 10.1111/j.1476-5381.2009.00278.x. 19508396 PMC2737650

[ref55] Suk J. S.; Xu Q.; Kim N.; Hanes J.; Ensign L. M. PEGylation as a strategy for improving nanoparticle-based drug and gene delivery. Adv. Drug Delivery Rev. 2016, 99, 28–51. 10.1016/j.addr.2015.09.012. PMC4798869 26456916

[ref56] Wood P. L.; Khan M. A.; Moskal J. R. The concept of ″aldehyde load″ in neurodegenerative mechanisms: Cytotoxicity of the polyamine degradation products hydrogen peroxide, acrolein, 3-aminopropanal, 3-acetamidopropanal and 4-aminobutanal in a retinal ganglion cell line. Brain Res. 2007, 1145, 150–156. 10.1016/j.brainres.2006.10.004. 17362887

[ref57] Radosavac D.; Graf P.; Polidori M. C.; Sies H.; Stahl W. Tocopherol metabolites 2, 5, 7, 8-tetramethyl-2-(2’-carboxyethyl)-6-hydroxychroman (α-CEHC) and 2, 7, 8-trimethyl-2-(2’-carboxyethyl)-6-hydroxychroman (γ-CEHC) in human serum after a single dose of natural vitamin E. Eur. J. Nutr. 2002, 41, 119–124. 10.1007/s00394-002-0365-3. 12111049

[ref58] van Meer G.; Voelker D. R.; Feigenson G. W. Membrane lipids: where they are and how they behave. Nat. Rev. Mol. Cell Biol. 2008, 9, 112–124. 10.1038/nrm2330. 18216768 PMC2642958

[ref59] Carrola J.; Bastos V.; Ferreira de Oliveira J. M.; Oliveira H.; Santos C.; Gil A. M.; Duarte I. F. Insights into the impact of silver nanoparticles on human keratinocytes metabolism through NMR metabolomics. Arch. Biochem. Biophys. 2016, 589, 53–61. 10.1016/j.abb.2015.08.022. 26344855

[ref60] Haase A.; Arlinghaus H. F.; Tentschert J.; Jungnickel H.; Graf P.; Mantion A.; Draude F.; Galla S.; Plendl J.; Goetz M. E.; Masic A.; Meier W.; Thünemann A. F.; Taubert A.; Luch A. Application of laser postionization secondary neutral mass spectrometry/time-of-flight secondary ion mass spectrometry in nanotoxicology: visualization of nanosilver in human macrophages and cellular responses. ACS Nano 2011, 5, 3059–3068. 10.1021/nn200163w. 21456612

[ref61] Saborano R.; Wongpinyochit T.; Totten J. D.; Johnston B. F.; Seib F. P.; Duarte I. F. Metabolic Reprogramming of Macrophages Exposed to Silk, Poly(lactic-co-glycolic acid), and Silica Nanoparticles. Adv. Healthcare Mater. 2017, 6, 1601240 10.1002/adhm.201601240. 28544603

[ref62] Batrakova E. V.; Kabanov A. V. Pluronic block copolymers: Evolution of drug delivery concept from inert nanocarriers to biological response modifiers. J. Controlled Release 2008, 130, 98–106. 10.1016/j.jconrel.2008.04.013. PMC2678942 18534704

[ref63] Nawaz S.; Redhead M.; Mantovani G.; Alexander C.; Bosquillon C.; Carbone P. Interactions of PEO-PPO-PEO block copolymers with lipid membranes: a computational and experimental study linking membrane lysis with polymer structure. Soft Matter 2012, 8, 6744–6754. 10.1039/c2sm25327e.

