# Supplementary material for: Metabolic Signatures of Surface-Modified Poly(lactic-co-glycolic acid) Nanoparticles in Differentiated THP-1 Cells Derived with Liquid Chromatography-Mass Spectrometry-based Metabolomics
Source: ACS Omega. 2022 Aug 12;7(33):28806–19. doi: 10.1021/acsomega.2c01660 (PMC9404530; doi:10.1021/acsomega.2c01660)
Supplement: Supplementary file 2 — ao2c01660_si_002.pdf [file ao2c01660_si_002.pdf]

Metabolic signatures of surface modified poly(lactic-co-glycolic acid) nanoparticles in differentiated THP-1 cells derived with LC-MS-based metabolomics

Mohammad A. Al-natour[1,4†], Salah Abdelrazig[2,5†], Amir M. Ghaemmaghami[3], Cameron Alexander[1], Dong-Hyun Kim[2*]

[†]Mohammad A. Al-natour and Salah Abdelrazig are joint first authors

[1]Molecular Therapeutics and Formulation Division, School of Pharmacy, University of Nottingham, NG7 2RD.

[2]Centre for Analytical Bioscience, Advanced Materials and Healthcare Technologies Division, School of Pharmacy, University of Nottingham, Nottingham, NG7 2RD, UK.

[3]Immunology&Immuno-bioengineering Group, School of Life Sciences, Faculty of Medicine and Health Sciences, Queen's Medical Centre, University of Nottingham, Nottingham, NG7 2RD, UK.

[4]Division of Pharmaceutics and Pharmaceutical sciences, Faculty of Pharmacy, University of Petra, Amman, Jordan

[5]Department of Pharmaceutical Chemistry, Faculty of Pharmacy, University of Khartoum, Khartoum, Sudan

*Corresponding author:

Dong-Hyun Kim
Centre for Analytical Bioscience, Advanced Materials and Healthcare Technologies Division
School of Pharmacy, University of Nottingham, Nottingham, NG7 2RD, UK
Email: dong-hyun.Kim@nottingham.ac.uk
Tel: +44 (0)115 748 4697

Table S1. Characterisation of the prepared NPs based on size measurement and zeta potential determined using Malvern Zetasizer

Type of NPs	Mean Diameter (nm) (±SD)	Zeta potential (mv) (±SD)
Plain PLGA NPs	101 ± 5	-43 ± 3
F-127 coated NPs	109 ± 6	-22 ± 4
F-68 coated NPs	105 ± 6	-33 ± 4
P85 coated NPs	102 ± 5	-36 ± 3
PEG-PLGA NPs	98 ± 7	-30 ± 5

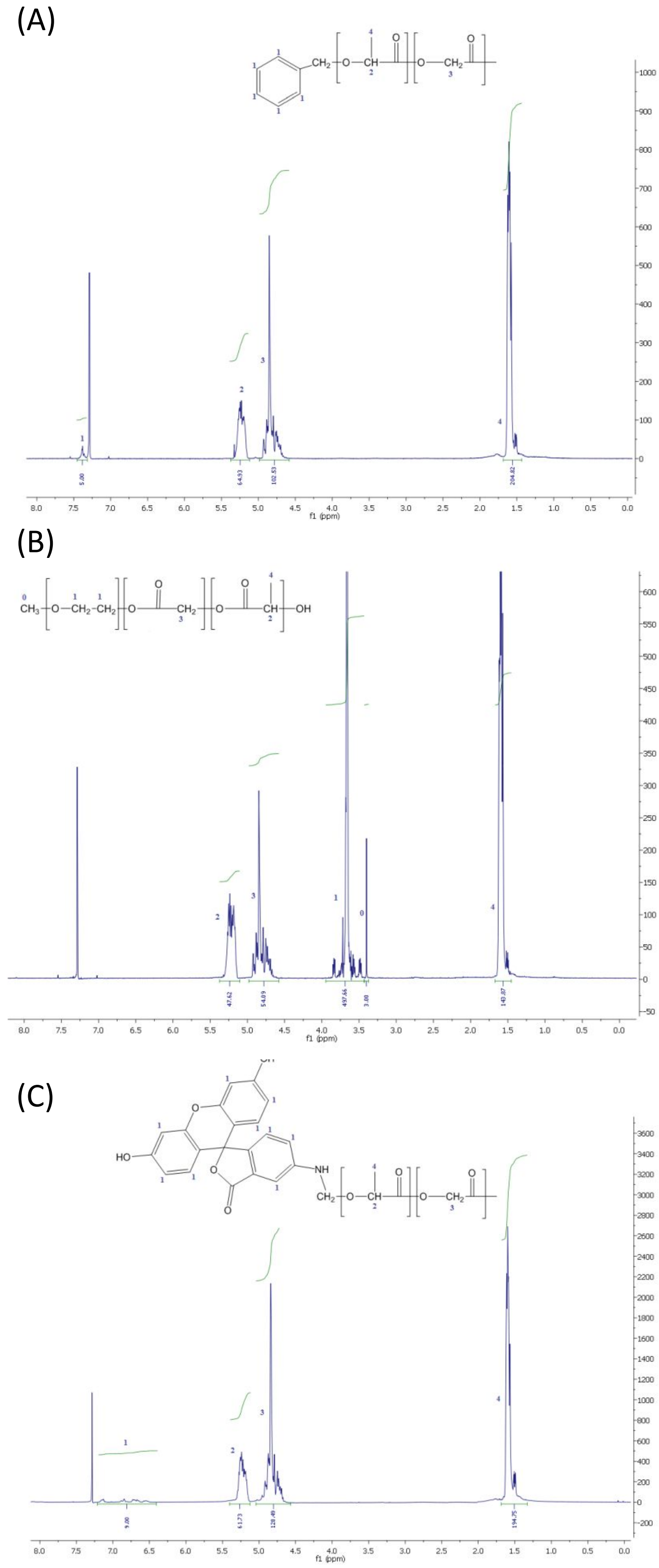

Figure S1. ^1H NMR spectra of (A) PLGA, (B) PEG-PLGA and (C) PLGA-5AF in $CDCl_3$.

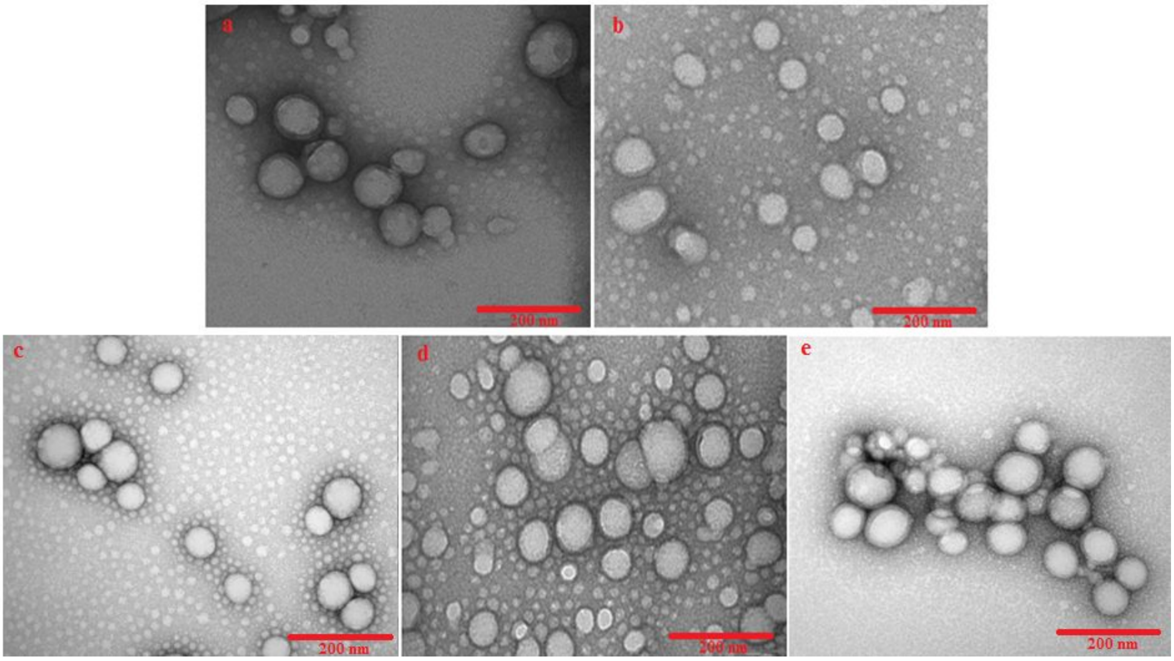

Figure S2. The morphology of the prepared NPs examined using Transmission Electron Microscopy (TEM). a, b, c, d and e present TEM micrographs of PLGA (plain), F-127, F-68, P85 coated PLGA and PEG-PLGA NPs.

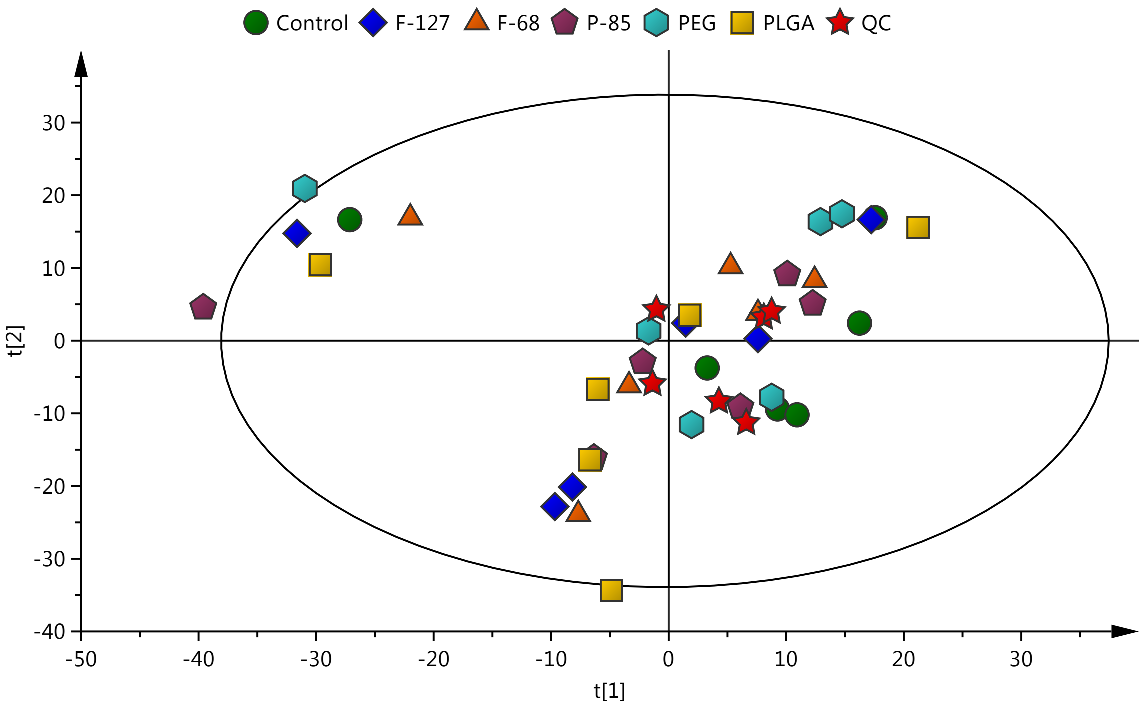

Figure S3. PCA scores plots of the metabolic profiles of the extract of the differentiated THP-1 cells after 24 h treatment with different types of NPs compared to control (no treatment) (cross validation: R^2X=0.700, Q^2=0.443)
